# Dissecting mechanisms of resistance to targeted drug combination therapy in human colorectal cancer

**DOI:** 10.1038/s41388-019-0780-z

**Published:** 2019-03-25

**Authors:** Paul A. Clarke, Toby Roe, Kate Swabey, Steve M. Hobbs, Craig McAndrew, Kathy Tomlin, Isaac Westwood, Rosemary Burke, Robert van Montfort, Paul Workman

**Affiliations:** Cancer Research UK Cancer Therapeutics Unit, The Institute of Cancer Research, London, SM2 5NG UK

**Keywords:** Colorectal cancer, Cell signalling, Molecular biology

## Abstract

Genomic alterations in cancer cells result in vulnerabilities that clinicians can exploit using molecularly targeted drugs, guided by knowledge of the tumour genotype. However, the selective activity of these drugs exerts an evolutionary pressure on cancers that can result in the outgrowth of resistant clones. Use of rational drug combinations can overcome resistance to targeted drugs, but resistance may eventually develop to combinatorial therapies. We selected MAPK- and PI3K-pathway inhibition in colorectal cancer as a model system to dissect out mechanisms of resistance. We focused on these signalling pathways because they are frequently activated in colorectal tumours, have well-characterised mutations and are clinically relevant. By treating a panel of 47 human colorectal cancer cell lines with a combination of MEK- and PI3K-inhibitors, we observe a synergistic inhibition of growth in almost all cell lines. Cells with *KRAS* mutations are less sensitive to PI3K inhibition, but are particularly sensitive to the combined treatment. Colorectal cancer cell lines with inherent or acquired resistance to monotherapy do not show a synergistic response to the combination treatment. Cells that acquire resistance to an MEK–PI3K inhibitor combination treatment still respond to an ERK–PI3K inhibitor regimen, but subsequently also acquire resistance to this combination treatment. Importantly, the mechanisms of resistance to MEK and PI3K inhibitors observed, MEK1/2 mutation or loss of PTEN, are similar to those detected in the clinic. ERK inhibitors may have clinical utility in overcoming resistance to MEK inhibitor regimes; however, we find a recurrent active site mutation of ERK2 that drives resistance to ERK inhibitors in mono- or combined regimens, suggesting that resistance will remain a hurdle. Importantly, we find that the addition of low concentrations of the BCL2-family inhibitor navitoclax to the MEK–PI3K inhibitor regimen improves the synergistic interaction and blocks the acquisition of resistance.

## Introduction

Over recent years an improved understanding of the molecular basis of cancer has led to the concept of precision medicine, where treatment with targeted drugs is guided by knowledge of the patient’s tumour genotype [1]. Although this approach can be successful, the selective pressure that targeted agents exert can result in the outgrowth of resistant clones [2–5]. The molecular responses of tumours to therapeutics targeting the MAPK pathway are paradigms for the development of resistance [3, 4, 6]. Early studies of BRAF- and MEK-inhibitors targeting this pathway established that they were particularly active in *BRAF* mutant melanoma [6–8]. However, even in this patient cohort, the response is generally temporary with most patients relapsing within a year [6]. To overcome resistance, an RAF–MEK inhibitor combination has been approved for the treatment of mutant *BRAF* melanoma and, more recently, has been shown to be effective in *BRAF* mutant colorectal cancer [9–12]. However, despite improved responses to these combination therapies tumours still recur [12, 13].

Colorectal cancers frequently have genetic aberrations in the MAPK or phosphatidylinositol 3-kinase (PI3K) pathways [14] and targeting these pathways can inhibit tumour growth [7, 15–17]. However, a significant proportion of colorectal tumours have genetic abnormalities that activate both pathways [14], resulting in a reduced response to monotherapy. The interconnectedness of these two pathways suggests that combination of MEK- and PI3K-inhibitors is a rational therapeutic approach. Several studies have explored combinations of MEK- and PI3K-inhibitors in vivo and in vitro and have reported both positive and negative effects of the combination treatment versus monotherapy [16, 18–21].

It is important to understand the mechanisms responsible for resistance to targeted cancer drugs to inform their future clinical use. In this study we focus on colorectal cancer and find that a combination of MEK- and PI3K-inhibitors is synergistic for growth inhibition across a panel of human colorectal cell lines, particularly in *KRAS* mutant cells with a reduced sensitivity to PI3K inhibition. We also find that prolonged exposure to a PI3K inhibitor plus an MEK- or ERK-inhibitor leads to the emergence of resistance; importantly, however, this resistance can be overcome by cotreatment with a BCL2-family inhibitor.

## Results

### MEK- and PI3K-inhibitors act synergistically in human colorectal cancer cells

**Fig. 1:**
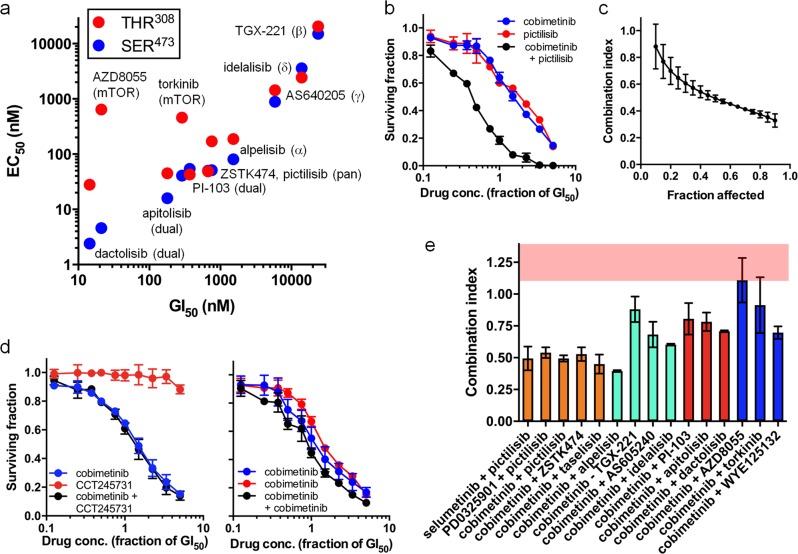
Synergistic inhibition of HCT116 human colorectal cancer cell growth by MEK and PI3K inhibitors. **a** Comparison of the EC_50_ for inhibition of AKT^SER473^ and AKT^THR308^ phosphorylation with the GI_50_ of PI3K inhibitors (EC_50_ values, *n* = 2) using an electrochemiluminescent ELISA. **b**, **c** Effect on HCT116 cell growth of 96 h exposure to pictilisib (pan-PI3K inhibitor) and cobimetinib (MEK inhibitor). **d** Effect of CCT245731 (an inactive analogue of pictilisib) and cobimetinib (Supplementary Tables S1-2) or single/repeated treatment with cobimetinib. **e** Combination indices for combination treatments featuring PI3K pathway inhibitors with different selectivity profiles. Column colours indicate PI3K pathway inhibitor selectivity: orange = pan-class I, cyan = class I isoform-selective, red = dual mTOR–pan-class I, blue = mTOR. The pink region indicates CI values associated with an additive effect, determined by combining two doses of cobimetinib (from (**d**)). Mean values (s.e.m.), *n* ≥ 3

Initially, we determined the potency of PI3K pathway inhibitors in a panel of 29 human colorectal cancer cell lines, in order to aid selection of exemplars for further analysis (Supplementary Table 1–2; Supplementary Fig. 1). To determine the relationship between inhibitor selectivity profile and its ability to block PI3K signalling, we also measured AKT phosphorylation in HCT116 human colorectal cancer cells (Fig. 1a), including additional PI3K pathway inhibitors to widen the analysis. For the majority of the inhibitors the EC_50_ values for inhibition of AKT^SER473^ and AKT^THR308^ phosphorylation are similar and correlate with GI_50_ values for cell growth inhibition (Fig. 1a). The mTOR-selective inhibitors and one of the dual mTOR–pan class I PI3K inhibitors, dactolisib, are an exception, with disparate EC_50_ values for inhibition of AKT^SER473^ and AKT^THR308^ phosphorylation (Fig. 1a).

We next used HCT116 and SW480 colorectal cancer cell lines, initially with the cobimetinib (MEK inhibitor; Supplementary Table 3) and pictilisib (class I PI3K inhibitor) to establish the combination methodology. We observe a synergistic interaction with CI values of 0.515 ± 0.030 and 0.305 ± 0.026 respectively at the combination EC_50_ (Fig. 1b, c; Supplementary Fig. 2a). As controls, substituting pictilisib with CCT245731 (an inactive analogue [15, 22]; Supplementary Table 1) eliminates the synergistic response (Fig. 1d), while a combination of cobimetinib with a second treatment of cobimetinib, predicted to give an additive value of 1, results in a measured CI of 1.241 ± 0.146 (Fig. 1d).

Having established that we could detect a synergistic interaction, we tested a selection of MEK inhibitors with different PI3K-pathway inhibitors in HCT116 and SW480 cells (Supplementary Tables 1 and 3). Combinations of all three MEK inhibitors, PD0325901, cobimetinib or selumetanib with pictilisib gave synergistic inhibition of cell growth (Fig. 1e). Similarly, all three pan-class I PI3K inhibitors, pictilisib, ZSTK474 and taselisib are equally synergistic with the MEK inhibitor cobimetinib. Alpelisib, which is highly selective for the alpha-isoform of PI3K, is the most effective of all of the isoform-specific PI3K inhibitors tested in combination with cobimetinib, producing effects equivalent to the pan-class I PI3K inhibitors. The dual mTOR–pan-class I PI3K inhibitors apitolisib, PI-103 and dactolisib are less effective than their pan-class I PI3K-selective counterparts when used in combination with cobimetinib. As previously described [21], we found that combining any of the mTOR-specific inhibitors, AZD8055, torkinib or WYE125132, with cobimetinib results in an additive, rather than synergistic, response in both lines (Fig. 1e; Supplementary Fig. 2b).

Based on their cell panel GI_50_ potency, activity against AKT phosphorylation and combination efficacy **(**Fig. 1; Supplementary Fig. 1–2**)**, we selected the pan-class I PI3K inhibitor pictilisib and the MEK inhibitor cobimetinib for use in subsequent experiments to explore the basis of resistance to combined MEK−PI3K inhibitor treatment.

### Combined MEK- and PI3K-inhibition induces apoptosis

**Fig. 2:**
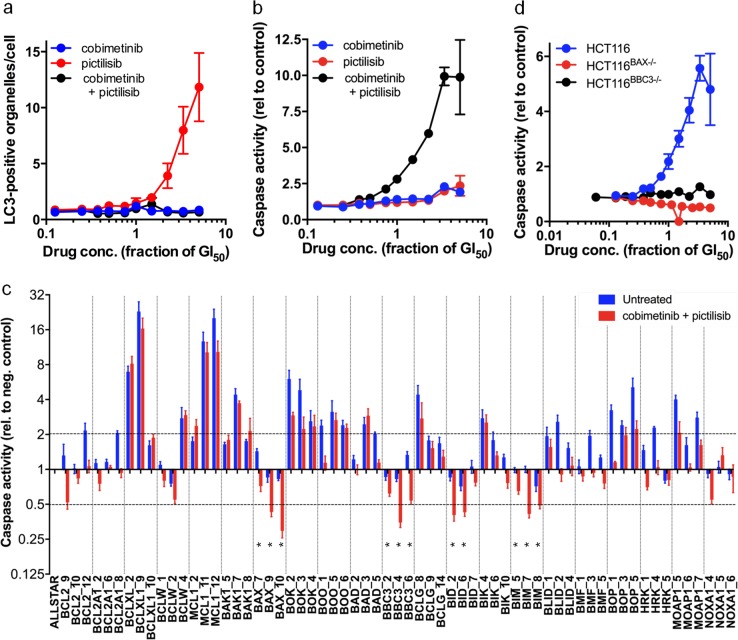
BAX, BBC, BID and BIM mediate apoptosis in HCT116 human colorectal cancer cells subjected to combined MEK–PI3K inhibition. Levels of **a** LC3 staining, indicative of autophagy, and **b** caspase activity, indicative of apoptosis, following 24 h treatment with cobimetinib and/or pictilisib. **c** Caspase 3/7 activity following transfection of cells with siRNA against proapoptotic genes for 48 h and subsequent treatment with 5× GI_50_ cobimetinib and pictilisib, or the vehicle control, for 24 h. Caspase activity is expressed relative to that in cells treated with Allstars negative control siRNA (*P ≤ 0.001). **d** Caspase activity in the HCT116 parental cells and HCT116^BBC3−/−^ or HCT116^BAX−/−^ cell lines after a combined treatment with cobimetinib and pictilisib. All plots show mean values (s.e.m.), *n* ≥ 3

Monotherapy with either cobimetinib or pictilisib does not induce apoptosis biomarkers, whereas monotherapy with the pictilisib alone increases the level of LC3B, potentially indicative of autophagy (Fig. 2a, b). In contrast, the combination treatment induces apoptosis-associated caspase activation in both HCT116 and SW480 cells with no detectable autophagy-associated LC3B signal (Fig. 2a, b; Supplementary Fig. 2c).

We used an siRNA minipanel to identify regulators of the apoptotic response induced by the combination regimen. At least 2/3 of the siRNAs targeting the expression of antiapoptotic *BCLXL* and *MCL1* induced apoptotic caspase activity. In contrast, siRNA silencing of proapoptotic genes *BIM*, *BBC3*, *BID* and *BAX* significantly reduces combination-induced apoptosis (Fig. 2c). We confirmed that BAX and BBC3 are required for the induction of apoptosis by the combination treatment using isogenic knockout HCT116^BAX−/−^ and HCT116^BBC3−/−^ cell lines (Fig. 2d) [23, 24]. Interestingly, the cobimetinib–pictilisib combination treatment retains its synergistic activity in both knockout lines (HCT116^BAX−/−^ CI = 0.518 ± 0.035, HCT116^BBC3−/−^ CI = 0.604 ± 0.035), indicating that apoptosis defined by caspase activation is not exclusively required for these inhibitors to act in synergy.

### Combined MEK- and PI3K-inhibition acts synergistically on RPS6 phosphorylation and Forkhead-regulated gene expression

**Fig. 3:**
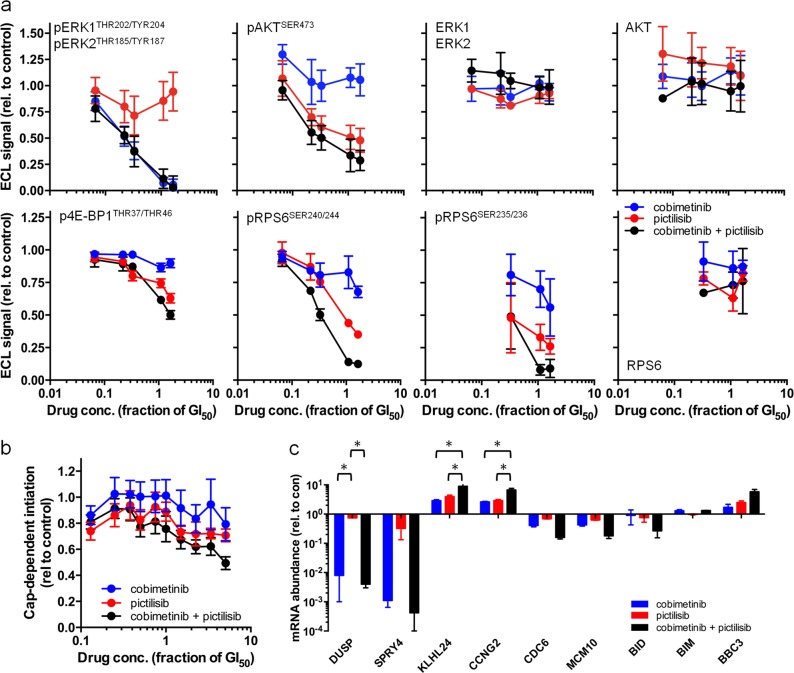
Combined MEK–PI3K inhibition has a synergistic effect on RPS6 phosphorylation and FOXO-regulated transcription in HCT116 human colorectal cancer cells. **a** Expression of phospho- or total proteins following 6 h exposure to cobimetinib and/or pictilisib. **b** Level of cap-dependent protein synthesis initiation (firefly luciferase) after 24 h exposure to cobimetinib and/or pictilisib. Data normalised to cap-independent protein synthesis initiation (EMCV IRES-driven *renilla* luciferase expressed from the same bicistonic mRNA) and expressed relative to vehicle control. **c** Transcript abundance determined by RT-PCR after 14 h exposure to cobimetinib and/or pictilisib (*P ≤ 0.001). All data are mean values (s.e.m) shown relative to that for cells treated with the vehicle control (*n* ≥ 3)

Next, we determined the molecular response to combined MEK- and PI3K-inhibition and observe a decrease in the levels of phosphorylated ERK and AKT (Fig. 3a; Supplementary Fig. S3a). However, the combination treatment is no more effective at inhibiting ERK or AKT phosphorylation than the single treatments. Similarly, the combination treatment is no more effective at inhibiting 4E-BP1 phosphorylation (Fig. 3a; Supplementary Fig. 3a) or global cap-dependent protein synthesis than monotherapy (Fig. 3b; Supplementary Fig. 3b). In contrast, we observe a significant synergistic reduction in the level of phosphorylated RPS6 following combination treatment, compared to levels after monotherapy (HCT116 CI = 0.268 ± 0.023; SW480 CI = 0.395 ± 0.066; *P* < 0.05; Fig. 3a; Supplementary Fig. 3a).

Levels of transcript for two Forkhead-regulated genes, *KLHL24* and *CCNG2* [25], are significantly increased by the combination treatment (Fig. 3c; Supplementary Fig. 4a). This contrasts with genes regulated by the individual PI3K (*CDC6* and *MCM10*) or MAPK (*DUSP6* and *SPRY4*) pathways [7, 25] or encoding proapoptotic factors (*BIM*, *BBC3* and *BID*) (Fig. 3d; Supplementary Fig. 4b-c). BBC3 and BIM are also induced at the protein level by pictilisib and cobimetinib monotherapy respectively, but not further induced by the combination (Supplementary Fig. 4b).

### MEK- and PI3K-inhibitor combination treatment has a synergistic effect in multiple colorectal cell lines

To determine CI values in our panel of 47 human colorectal cancer cell lines we first established GI_50_ values for cobimetinib, pictilisib or the dual mTOR–pan-class I PI3K inhibitor apitolisib (Supplementary Fig. 5). We observe that *KRAS-*mutant *PIK3CA*-wild-type cells are significantly less sensitive to the PI3K inhibitor pictilisib than the wild type (*P* = 0.019, *q* = 0.0498) or *KRAS*-wild-type *PIK3CA*-mutant cells (*P* = 0.0076, *q* = 0.0384). Cells with *KRAS* codon 12 mutations (*KRAS*^CDN12^/PIK3CA^WT^) are the least sensitive to PI3K inhibition overall (*P* = 0.0051, *q* = 0.0108; Supplementary Fig. 5). Most *BRAF* mutant cell lines are very sensitive to the MEK inhibitor cobimetinib compared to wild-type cells (BRAF^MUT^ *P* = 0.0487, *q* = 0.0804; BRAF^MUT^/PIK3CA^MUT^ *P* = 0.0085, *q* = 0.0281).

**Fig. 4:**
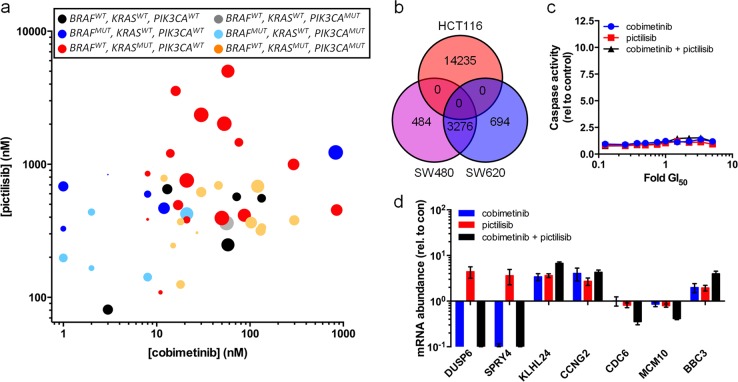
Combined MEK–PI3K inhibitor treatment is synergistic across a human colorectal cancer panel. **a** GI_50_ values for pictilisib versus cobimetinib in each cell line, determined after a 96 h treatment; data point size denotes the CI value, range: 0.296−0.884 (inverse correlation). **b** Venn diagram showing the number of SNVs detected in HCT116, SW480, and SW620 human colorectal cancer cell lines that are predicted to alter protein sequence. **c** Levels of caspase 3/7 activity, indicative of apoptosis, in SW620 cells exposed to pictilisib or cobimetinib for 24 h. **d** Transcript abundance in SW620 cells exposed to pictilisib and/or cobimetinib for 6 h. Mean levels (s.e.m.) are shown relative to those in cells treated with a vehicle control (*n* ≥ 3)

We find that the interaction between cobimetinib and pictilisib is synergistic in all but one colorectal cell line, with CI values ranging from 0.296 to 0.884 (Fig. 4a; Supplementary Table 4). There is a strong synergistic response (CI < 0.5) to the combination treatment in many *KRAS* mutant lines, but this is not statistically significant across the whole panel (Supplementary Fig. 6a). As observed with the HCT116 and SW480 cell lines, the synergistic effect of the combination treatment is reduced when the dual mTOR–PI3K inhibitor apitolisib is used in place of pictilisib (Supplementary Fig. 6b).

The growth of COLO320 cells is insensitive to MEK inhibition (cobimetinib GI_50_ = 1455 nM; Supplementary Table 4) and shows no evidence of synergy with the combination. We also find that SW620 cells (lymph node metastasis)—which are derived from the same patient as SW480 cells (primary tumour) (Fig. 4b)—are less sensitive to pictilisib (Supplementary Fig. 6c) and also to the cobimetinib–pictilisib combination (SW480 CI = 0.310 ± 0.018, SW620 CI = 0.596 ± 0.017, *P* < 0.001). Consistent with this, the combined MEK–PI3K inhibitor treatment of SW620 cells does not induce apoptosis and has no additional effect on RPS6 phosphorylation or Forkhead-regulated gene expression (Fig. 4c, d; Supplementary Figs. 2c, 3, 4a and 6c).

Overall, across the large panel of colorectal cell lines with a range of genetic backgrounds, combined MEK- and PI3K-inhibition is highly synergistic, particularly in *KRAS* mutant cancers. In addition, we find that inherent resistance or reduced sensitivity to the MEK−PI3K inhibitor combination is rare, but can result from reduced or loss of sensitivity to either arm of the combination therapy.

### Cells with acquired insensitivity to MEK- or PI3K-inhibitor monotherapy are also resistant to combination therapy

**Fig. 5:**
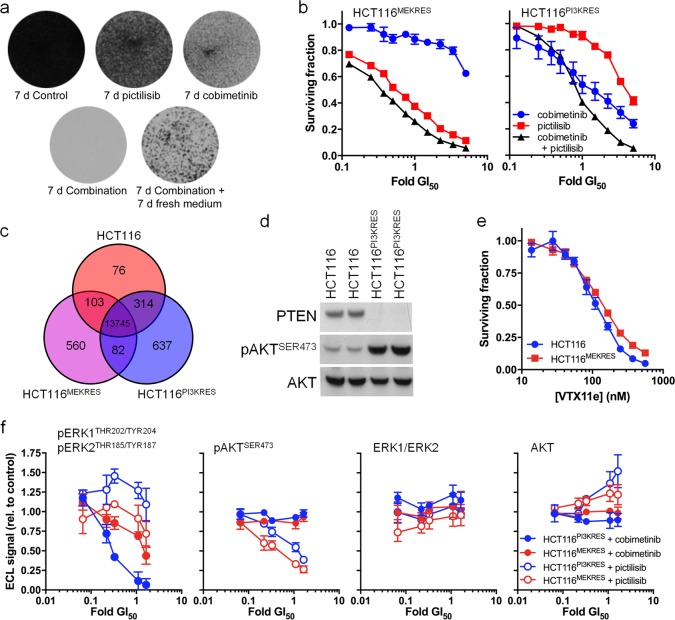
HCT116 human colorectal cancer cells resistant to MEK or PI3K inhibitor monotherapy are insensitive to combination treatment. **a** Photographs of HCT116 cells treated with 5× GI_50_ cobimetinib and/or pictilisib. **b** Fraction of HCT116^MEKRES^ or HCT116^PI3KRES^ cells surviving 96 h exposure to pictilisib and/or cobimetinib. **c** Venn diagram showing the number of SNVs or INDELs predicted to alter protein sequence in HCT116, HCT116^MEKRES^ and HCT116^PI3KRES^ cells. **d** Immunoblot indicating the abundance of PTEN, pAKT^SER473^ and total AKT in two independent cultures of HCT116 and HCT116^PI3KRES^ cells. **e** Fraction of cells surviving 96 h exposure to VTX11e. **f** Mean levels (s.e.m.) of pERK1^THR202/TYR204^ and pERK2^THR185/TYR187^, pAKT^SER473^, total ERK1/2 and total AKT after 6 h exposure to drug or a vehicle control (*n* ≥ 3)

We found that some cells that survived 7 days exposure to the combination treatment were able to proliferate when fresh drug-free medium was added (Fig. 5a). To dissect out potential resistance mechanisms, we first examined cells with acquired resistance to the individual elements of the combined regimen. Exposure of HCT116 cells to increasing concentrations of MEK or PI3K inhibitors over a period of 8–10 weeks generated an MEK inhibitor resistant line (HCT116^MEKRES^) with a GI_50_ for cobimetinib (401 ± 55 nM) 13-fold greater than that of the parental line and also a PI3K inhibitor resistant line (HCT116^PI3KRES^) with a GI_50_ for pictilisib (3119 ± 266 nM; Fig. 5b) 5-fold greater than that of the parent line. HCT116^MEKRES^ cells fail to show a synergistic response to the combination treatment, even when the concentration of cobimetinib used is increased (Fig. 5b). Treating HCT116^PI3KRES^ cells with a concentration of cobimetinib and pictilisib that is effective on the parental cells does not result in a synergistic inhibition of cell growth but, in contrast to the HCT116^MEKRES^ cells, there is a synergistic response to the combination treatment when a fivefold higher concentration of pictilisib is used (CI = 0.589 ± 0.026; Fig. 5b).

Whole-exome sequencing showed that >92% of the single nucleotide variants (SNV) and insertions or deletions (INDELS) were conserved between the parent and resistant cells, confirming that the two resistant lines were derived from parent HCT116 cells (Fig. 5c). Among the variants unique to the HCT116^PI3KRES^ cells are two frameshift mutations in *PTEN* (*PTEN*^K14fs*^ and *PTEN*^N323fs*^). Sequencing of *PTEN* cDNA clones isolated from HCT116^PI3KRES^ cells detected only mutant cDNAs (Supplementary Fig. 7), consistent with the loss of PTEN protein and elevated phosphorylation of AKT^SER473^ (Fig. 5d) that still remains sensitive to pictilisib treatment at high concentrations (Fig. 5f), explaining the synergistic response to the combination treatment at high pictilisib concentrations.

Exome sequencing of HCT116^MEKRES^ identified a MEK1^L215P^ mutation previously detected in other MEK inhibitor resistant cell lines or patients that prevents MEK inhibitor binding [26]. These HCT116^MEKRES^ cells are cross-resistant to the MEK inhibitors selumetanib (GI_50_ > 2000 nM) and trametinib (Supplementary Fig. 8a and Supplementary Table 3) but remain sensitive to the ERK inhibitor VTX11e [27] and to VTX11e–pictilisib combination therapy (CI = 0.551 ± 0.028), indicating that the MEK–ERK pathway is still required by HCT116^MEKRES^ cells (Fig. 5e). ERK1/2 phosphorylation is less responsive to cobimetinib treatment in the HCT116^MEKRES^ cell line, consistent with the inability of cobimetinib to bind mutated MEK (Fig. 5f).

### Sustained combination treatment results in the acquisition of resistance

**Fig. 6:**
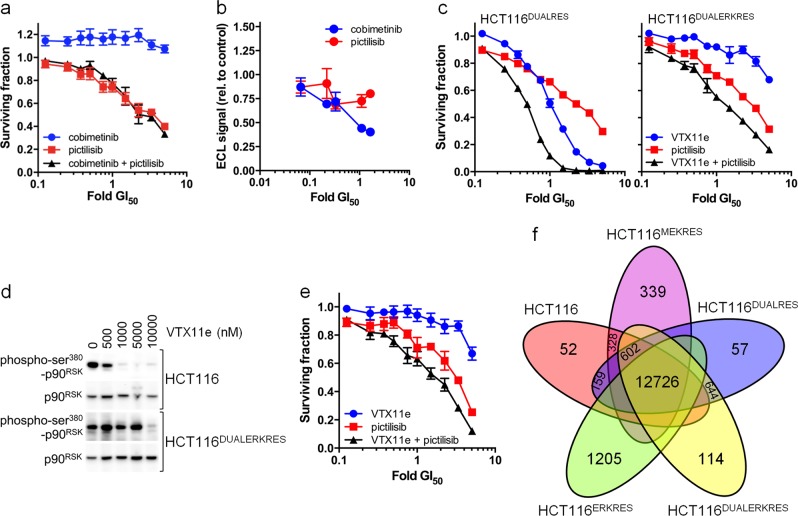
Characterisation of HCT116 human colorectal cancer cells with acquired resistance to MEK or ERK inhibitor-based combination therapy. **a** Surviving fraction of HCT116^DUALRES^ cells treated with pictilisib and/or cobimetinib for 96 h. **b** Level of pERK1^THR202/TYR204^/pERK2^THR185/TYR187^ in HCT116^DUALRES^ cells exposed to pictilisib or cobimetinib for 6 h. Data are shown relative to levels in cells treated with a vehicle control. **c** Surviving fraction of HCT116^DUALRES^ and HCT116^DUALERKRES^ cells after 96 h exposure to pictilisib and VTX11e. **d** Levels of total and phospho-SER^380^ p90^RSK^ following treatment of HCT116 and HCT116^DUALERKRES^ with VTX11e for 6 h. **e** Surviving fraction of HCT116^ERKRES^ cells after 96 h treatment with pictisilib and/or VTX11e. **f** Venn diagram showing the number of SNVs or INDELs predicted to alter protein sequence in HCT116, HCT116^MEKRES^, HCT116^DUALRES^, HCT116^DUALERKRES^ and HCT116^ERKRES^ cells. For all plots *n* ≥ 3 and error bars indicate s.e.m.

We next cultured HCT116 colorectal cancer cells in increasing concentrations of cobimetinib and pictilisib and find they acquired resistance to the combination treatment over a period of 8–10 weeks. This resistance is due to reduced sensitivity to MEK inhibition (Fig. 6a, b). As observed for HCT116^MEKRES^, we find that the combination-resistant HCT116^DUALRES^ cells are cross-resistant to selumetanib (GI_50_ > 2000 nM) and trametinib (Supplementary Fig. 8a). Replacing the MEK inhibitor cobimetinib arm of the combination treatment with the ERK inhibitor VTX11e (Fig. 6c) restores synergy (CI = 0.693 ± 0.034 nM), indicating that a dependence on signalling downstream of MEK still remains. Importantly, exposure of the HCT116^DUALRES^ line to increasing concentrations of VTX11e and pictilisib subsequently resulted in the acquisition of resistance to this combination treatment as a result of loss of sensitivity to VTX11e at the level of ERK1/2 (Fig. 6c, d). This cell line (HCT116^DUALERKRES^) retains resistance to the original cobimetinib–pictilisib combination (Supplementary Fig. 8b). Subsequently, we generated HCT116 cells with acquired resistance to ERK inhibitor monotherapy (HCT116^ERKRES^; Fig. 6f), which unlike the HCT116^DUALERKRES^ line, is sensitive to MEK inhibition—consistent with derivation from the parent HCT116 cells (Supplementary Fig. 8c).

Exome sequencing confirmed that the combination- and VTX11e-resistant lines (HCT116^ERKRES^, HCT116^DUALRES^ and HCT116^DUALERKRES^) are also derived from parental HCT116 cells (Fig. 6f). HCT116^DUALRES^ had acquired an MEK2^V215E^ mutation that prevents inhibitor binding, analogous to that detected in HCT116^MEKRES^ cells. We found that HCT116^DUALERKRES^ also shared 644 variants with HCT116^DUALRES^ cells, including the MEK2^V215E^ mutation, consistent with its derivation from HCT116^DUALRES^. In addition, HCT116^DUALERKRES^ cells acquired 114 unique variants including an ERK2^Y36H^ mutation. The ERK2^Y36H^ mutation is also detected in the HCT116^ERKRES^ cells. Consistent with their derivation from the parent cells, the HCT116^ERKRES^ cells lack the MEK2^V215E^ mutation and the additional sequence variants found in HCT116^DUALRES^ and HCT116^DUALERKRES^ cells.

### The Y36H mutant found in ERK inhibitor resistant cells is insensitive to ERK inhibitors

**Fig. 7:**
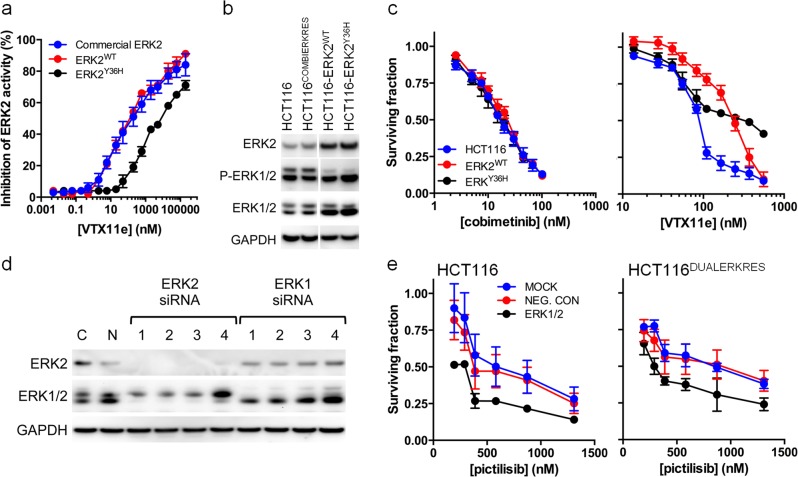
The ERK2^Y36H^ mutation does not affect MAPK-pathway signalling but induces resistance to small molecule ERK inhibitors. **a** Effect of VTX11e on ERK2 kinase activity. **b** Abundance of ERK2, pERK1/2 and total ERK1/2 in the parental HCT116 line, drug combination-resistant HCT116^DUALERKRES^ line and recombinant lines overexpressing ERK2^WT^ or ERK2^Y36H^. **c** Fraction of HCT116 and ERK2-overexpressing cells surviving 96 h treatment with cobimetinib or VTX11e. **d** Level of ERK2 and ERK1/2 in cells transfected with siRNA targeting *ERK1/2* expression (C = mock control, N = Allstars negative control nontargeting siRNA; ERK1 siRNA nos. 6, 7, 8, 11 and ERK2 siRNA nos. 10, 11, 12, 13; Qiagen, Germany). **e** Fraction of HCT116 or HCT116^DUALERKRES^ cells surviving 96 h exposure to pictilisib after siRNA knockdown of *ERK1/2* expression for 48 h prior to drug treatment. For all data *n* = 3 and, where applicable, error bars indicate s.e.m. GADPH was used as a loading control in immunoblots

Next, we determined the functional impact of the ERK2^Y36H^ mutation. The values of *K*_m_ and *V*_max_ found for ATP corresponding to ERK2^WT^ and ERK2^Y36H^ enzymes are similar (Supplementary Fig. 9a). ERK2^Y36H^ exhibited 19-fold resistance to VTX11e, 5-fold resistance to the ERK inhibitor ravoxertinib [28] and 2.7-fold resistance to SCH772984, a chemically distinct ERK1/2 inhibitor (Fig. 7a, Supplementary Fig. 9a-b; Supplementary Table 5) [29]. The differential resistance of the ERK2^Y36H^ protein to these inhibitors reflects the activity of the inhibitors in HCT116^DUALERKRES^ and HCT116^ERKRES^ cells, with a hierarchy of fold-resistance in the order of VTX11e > ravoxertinib > SCH772984 (Supplementary Fig. 10). HCT116^DUALERKRES^ and HCT116^ERKRES^ cells are also resistant to ulixertinib, a VXT11e derivative undergoing clinical studies, but not to the mechanistically distinct DEL-22379, which targets ERK dimerisation (Supplementary Fig. 10) [30–32].

Modelling of the ERK^Y36H^ mutation using published X-ray structures suggests that repulsion between the electron-dense imidazole ring of H36 and the partial negative charge of the aryl chlorine in VTX11e, also found in ulixertinib and ravoxertinib, predicted to make binding to ERK2^Y36H^ unfavourable (Supplementary Fig. 11a-c) [27, 28, 33]. The aryl chlorine is essential for the selectivity of VTX11e for ERK2 over other kinases (Supplementary Table 2). In contrast, this mutation is predicted to have little effect on the binding of SCH772984 to ERK2, as both Y36 or H36 are predicted to fold under the active site P-loop (Supplementary Fig. 11d-e), explaining why ERK2^Y36H^ cells are only slightly less sensitive to SCH772984 than wild type.

Exogenous expression of ERK2^WT^ or ERK2^Y36H^ results in the parent HCT116 cells increased ERK2 phosphorylation, but decreased phosphorylation of endogenous ERK1 (Fig. 7b). Cells expressing exogenous ERK2^WT^ or ERK2^Y36H^ retain their sensitivity to MEK inhibition by cobimetinib (Fig. 7c). Expression of ERK2^WT^ slightly increases the VTX11e GI_50_, consistent with ERK2 amplification driving ERK inhibitor resistance [34] (HCT116, 113 ± 15 nM; HCT116-ERK2^WT^, 198 ± 30 nM); in contrast, cells expressing ERK2^Y36H^ are resistant to VTX11e (GI_50_ > 500 nM; Fig. 7c). Simultaneous depletion of *ERK1/2* mRNA using siRNA affects the growth of HCT116 and HCT116^DUALERKRES^ cells equally and sensitised both lines to cotreatment with pictilisib (Fig. 7d, e). This indicates that the HCT116^DUALERKRES^ line remains susceptible to combined inhibition of MEK/ERK and PI3K, consistent with acquired resistance mutations in ERK2 and MEK2 that affected inhibitor binding but not enzymatic activity.

### BCL2-family inhibitor blocks the acquisition of resistance to MEK–PI3K combination treatment

**Fig. 8:**
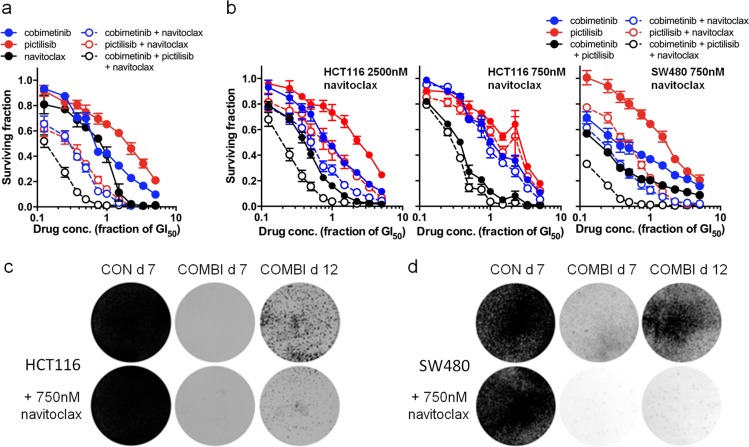
Navitoclax administration prevents cells acquiring resistance to a pictilisb–cobimetinib combination treatment. **a** Three-way combination treatment of HCT116 cells with cobimetinib, pictilisib and navitoclax at 1:1:1 ratios of their respective GI_50_s. **b** Combination treatment of HCT116 and SW480 cells with cobimetinib and pictilisib at a 1:1 ratio of their respective GI_50_s ± a low fixed concentration of navitoclax. For all plots (**a**, **b**) data are mean (s.e.m.) values, *n* ≥ 3. **c**, **d** Photographs of **c** HCT116 and **d** SW480 cells treated with 5× GI_50_ pictilisib–cobimetinib (combination treatment) or the combination treatment ± 750 nM navitoclax. Cells were seeded in duplicate six-well plates for 2 d before 7 d drug treatment or 7 d drug treatment plus a further 5 d in drug-free media

BCL2-family inhibitors can act synergistically with MEK- or PI3K-inhibitors [35–37], and also block the acquisition of resistance to MEK inhibition [38]. We find that *BCLXL* siRNA induced apoptosis when combined with pictilisib and cobimetinib (Fig. 2c). These observations prompted us to expose HCT116 cells to a cobimetinib−pictilisib combination treatment plus the BCL2-family inhibitor navitoclax. Cotreatment with navitoclax (GI_50_ = 7362 ± 247 nM) improves the CI for pictilisib plus cobimetinib in HCT116 cells (CI = 0.406 ± 0.037), potentially by acting in synergy both with pictilisib (CI = 0.552 ± 0.080) and cobimetinib (CI = 0.636 ± 0.042; Fig. 8a). This effect was less pronounced when pictilisib was replaced with the dual mTOR/pan-class I PI3K inhibitor apitolisib (Supplementary Fig. 12a). We also found that addition of a fixed concentration of navitoclax increases sensitivity to the cobimetinib–pictilisib combination in HCT116 cells (2500 nM navitolcax CI = 0.290 ± 0.021; 750 nM navitoclax CI = 0.387 ± 0.043; Fig. 8b). HCT116^DUALRES^ cells with previously acquired resistance to the combined MEK−PI3K inhibitor regimen through loss of sensitivity to MEK inhibition retained the synergistic interaction between pictilisib and navitoclax, but that was not further improved by the addition of cobimetinib (Supplementary Fig. 12b). In the HCT116^DUALRES^ cells, replacement of cobimetinib with the ERK inhibitor VTX11e rescued the potent synergistic activity of the triple combination, but again synergy was lost in HCT116^DUALERKRES^ cells with previously acquired resistance to the ERK inhibitor combination regimen (Supplementary Fig. 12c-e). Importantly, in longer-term clonogenic assays, we find that cotreatment with 750 nM navitoclax reduced the number of HCT116 and SW480 cells surviving 7 days exposure to the cobimetinib–pictilisib combination, such that we were unable to generate stable clones with acquired resistance in longer-term cultures (Fig. 8c, d).

## Discussion

Combination treatment regimens have several advantages over monotherapy: they may increase antitumour effects within acceptable toxicity limits, are more likely to be effective against a heterogeneous tumour population and may delay or block the development of drug resistance [39]. Here, we used a panel of human colorectal cancer cell lines to explore response to mono- and combination-therapies targeting MEK and PI3K. Initial sensitivity profiling work was followed up with a focused strategy using a single colorectal cancer cell line model to identify mechanisms of resistance. While the latter approach may not define the full spectrum of resistance in colorectal cancer, we did find resistance mechanisms, namely loss of PTEN and mutation of MEK1/2, that were previously reported in clinical resistance to MEK and PI3K inhibitors [26, 40], thus validating the approach.

Our results indicate that MEK–PI3K inhibitor combination treatment affects multiple nodes at the intersection of the MAPK and PI3K signalling pathways; these include reduced RPS6 phosphorylation and induced Forkhead-regulated gene expression [25]. RPS6 is phosphorylated by p70 and p90 S6 kinases, which are themselves regulated by MAPK and PI3K signalling, while the localisation and stability of the Forkhead transcription factors is regulated by AKT and ERK-mediated phosphorylation respectively [41–43]. Whether or not the overall synergistic effect of combined MEK- and PI3K-inhibition requires activity at multiple nodes, or just at one critical node, remains to be determined. We also find that BIM, BBC3 and BAX are required for the combination treatment to induce apoptosis, in line with previous studies [35, 44]; however, apoptosis measured by apoptotic protease activation is itself not essential for synergy since we observe that isogenic BBC3 or BAX knockout cells retained the synergistic growth inhibition response to MEK−PI3K inhibitors in the absence of caspase activation.

We find the selectivity profile of the PI3K inhibitor impacts the effectiveness of the combination regimen, as mTOR, dual mTOR–PI3K inhibitors and PI3K-inhibitors lacking activity against PIK3CA or poor potency against AKT^THR308^ phosphorylation have reduced synergy in combination with MEK inhibition. The consistency of these observations across multiple PI3K inhibitors, the majority of which have been clinically tested, suggests that other PI3K pathway inhibitors that can inhibit the class I alpha-isoform but lack MTOR inhibitory activity, such as copanlisib recently licensed for the treatment of follicular lymphoma [45], will have a similar activity profile in combination regimes. Almost all of the 47 human colorectal cancer cell lines tested exhibit a synergistic response to the optimal MEK−PI3K inhibitor combination treatment. However, colorectal cancer cell lines with inherent or acquired resistance to either of the treatment arms exhibit a reduced synergistic response to the combination regimen. Importantly, by exposing sensitive parent HCT116 cells to combination treatments for a prolonged period we generated cell lines that had lost the synergistic response that was initially exhibited to the MEK–PI3K combination, and subsequently also to the ERK–PI3K inhibitor combination that was at first able to overcome the resistance to the original MEK–PI3K combination. In both cases, loss of synergy is associated with the appearance of MEK and ERK mutations that were predicted or shown to disrupt inhibitor binding [26, 46] without overtly affecting MEK/ERK activity.

In contrast, resistance to PI3K inhibitor monotherapy was found to involve pathway deregulation through loss of PTEN, which we found can be overcome with higher concentrations of PI3K inhibitor. The absence of mutations preventing inhibitor binding to the PIK3CA lipid kinase domain may reflect the fact that these types of mutation are not well tolerated, because they tend to also inhibit enzyme activity [47]. This suggests that combination resistance-inducing mutations preferentially occur in MEK1/2 or ERK2 because mutations that disrupt inhibitor binding are better tolerated in these targets and do not affect enzymatic activity. Alternatively, or in addition, the parent HCT116 cell line used in this study is highly dependent on MAPK signalling, and disrupting this pathway has a greater impact on the efficacy of the combination regimen.

ERK inhibitors are in early clinical studies, including trials to determine if these agents can rescue resistance to BRAF- and MEK-inhibitors [32, 48, 49]. In addition to our own observations, studies expressing exogenously mutated forms of ERK, or exposing cell lines to ERK inhibitors, have identified mutations associated with resistance to ERK inhibitors [34, 50, 51]. Interestingly, a recent computational approach to identify resistance mutations likely to arise following drug treatment predicts mutation of ERK2^Y36^ as one of a number of mutation hotspots generating resistance to ERK inhibition [52]. Together, those studies and our own data suggest that resistance to ERK inhibitors may be inevitable in the clinic and that switching the inhibitor may delay, but will not avoid, the emergence of resistance.

Overall, we clearly show that a combination treatment targeting the MEK- and PI3K-pathways is insufficient to block the acquisition of resistance in human colorectal cancer cell line models, but the addition of a third agent, a BCL2 inhibitor, is able to do this. Several studies have shown that addition of a third agent can overcome acquired resistance or enhance the combinatorial effect of single agent MAPK- and PI3K-inhibitor regimens [53–55]. These observations suggest that the addition of a third agent, in our case a BCL2-family inhibitor, to create triple combinations may generate a fitness threshold that is too high to surpass by resistance mechanisms involving gene mutation or amplification. Importantly, the synergistic effect of the three-way combination was lost in cells with prior acquisition of resistance to one arm of the combination, suggesting that the triple therapy strategy is better suited to preventing the acquisition of resistance rather than overcoming existing intrinsic or acquired resistance from prior treatment. Preclinical studies have established a strong rationale for combined inhibition of PI3K- and MAPK-pathways and numerous early clinical studies exploring this approach are either in progress or completed [56]. Results presented as meeting abstracts generally report only modest activity of the combinations, often limited by toxicity [56]. Hence the addition of a third agent, while potentially beneficial, would require careful consideration of tolerability. It is feasible that short exposures to higher concentrations may have similar or greater antitumour activity while being better tolerated than continuous exposures to lower doses. For example, short intermittent exposures to a MEK-PI3K inhibitor combination treatment are sufficient to drive an in vivo response in preclinical models [16, 53]. Thus it is possible that the addition of a third agent may be tolerated clinically in such a dosing regimen and provide the opportunity to overcome or ameliorate the major clinical of clinical problem of resistance to targeted therapies.

## Materials and methods

### Compounds

Compounds were synthesised in-house or purchased from AxonMedChem (Groningen, Netherlands), Active Biochem (Kowloon, Hong Kong), Selleckchem (Boston, Massachesetts, USA) or Tocris (Bristol, UK).

### Cell culture models, assays and treatment

All cell lines were obtained from accredited cell banks, validated by DNA profiling and confirmed free of *Mycoplasma* spp. by PCR. HCT116^BAX−/−^, HCT116^BBC3−/−^ and parent isogenic cell lines were obtained from Horizon Discovery (UK). GI_50_ values (concentration that inhibits cell proliferation by 50%) were determined by sulphurhodamine blue staining [57]. Combination indices (CI) were determined using median effect analysis [58]. Cells were seeded in 96-well plates and at 2 days test compounds were added at a ratio of 1:1 of their respective GI_50_ values. After a further 4 days, cells were sulphurhodamine blue stained and the CI calculated using Calcusyn (Biosoft, UK). A CI value of 1 indicates an additive effect, <1 synergy and >1 antagonism. Longer term colony growth assay experiments were performed in six-well plates. At the end of the experiment cells were fixed and stained using Crystal Violet. Apoptosis was quantified by assaying cleavage of a fluorescent caspase 3/7 substrate (Promega, USA) and autophagic cells were quantified using an LC3B immunofluorescence assay (Thermo Fisher Scientific, USA) [59].

Stable resistant lines were generated by incremental exposure to inhibitors. Cells were passaged when they reached confluence and compound exposure increased until the concentration reached 64 × GI_50_ or the solubility limit.

Reporter cells were generated by transfection with a plasmid encoding a cap-dependent firefly luciferase and an EMCV IRES-driven *Renilla* luciferase (Promega, USA) downstream of an EF1α promoter [60]. *ERK2*^*WT*^ or *ERK2*^*Y36H*^ open reading frames were cloned into the expression vector pEFIRES-P [60]. Cells were transfected using lipofectamine (Thermo Fisher Scientific, USA) and cells with stable vector incorporation selected with puromycin.

Cells were reverse transfected with targeting siRNAs or positive or negative control siRNAs (Qiagen, Germany) using hiperfect cationic lipid (Qiagen, Germany). Cells were transfected in 96-well plates for viability assays or six-well plates to confirm the effects on target protein expression.

### Analysis of gene expression

mRNA abundance was determined by RT-PCR using a 7900HT Fast Real-Time PCR System (Applied Biosystems, USA). Levels of phosphorylated and total protein were investigated by immunoblotting or ELISA (Mesoscale Discovery, USA) [17, 59]. Antibodies were obtained from Cell Signaling Technologies (AKT^SER473^-#4060, total-AKT-#4691, BIM-#12450, BBC3-#2933, ERK1/2^THR202/TYR204^-#9101, total-ERK1/2-#9102, p90RSK^SER380^-#11989, total-p90RSK-#9355, PTEN-#5385; USA), or Merck (ERK2-#06–333, GAPDH-#CB1001; Germany). EC_50_ values were calculated from the ELISA data as the concentration that reduced protein phosphorylation by half.

### Next-generation whole-exome sequencing

Genomic DNA was extracted (Qiagen, Germany) and exome capture performed using the SureSelect Human All Exon V5 kit (Agilent, USA). Products were sequenced using a paired end sequencing protocol (v1.5) on a HiSeq 2500 (Illumina, USA). Reads were aligned to the human genome reference sequence GRCh37 using bwa [61]. Optical and PCR duplicates were marked in BAM files using Picard 1.107 (http://picard.sourceforge.net), additional BAM file manipulations were performed using Samtools (v0.1.18) and variant calling performed with FreeBayes (v0.9.20–8) [62]. Data are available on the NCBI Sequence Read Archive (SRA; https://www.ncbi.nlm.nih.gov/sra) website under accession number SUB4997621.

### ERK2 protein purification and biochemical assay

To generate active ERK2^WT^ or ERK2^Y36H^, GST-ERK2 (pCDFDuet-1) was coexpressed with constitutively active MEK1^S218E/S222E^ (pET-30b; Merck Chemicals Ltd, Germany) in BL21-AI™ *E. coli* (Thermo Fisher Scientific, USA). Active protein was purified over a GSTrap™ FF column and digested with HRV 3C protease. Cleaved protein was purified with a HiLoad 16/600 Superdex 200 pg column in series with 2 × 5 ml GSTrap™ FF columns followed by a Mono Q® HR 5/5 column. All chromatography columns were from GE Healthcare (UK).

ERK2 kinase activity was determined using an EZ Reader II (PerkinElmer, USA). Conversion to product, and inhibition, was calculated relative to no enzyme and all reagent conditions. IC_50_ values (concentration that reduced substrate conversion by half) were calculated using a four-parameter logistics fit (Dotmatics, UK).

### Statistical analysis

Normal data distribution was determined using a D’Agostino and Pearson normality test. Significance for normally distributed data was determined with a one-way ANOVA and *t* test analyses and for non-normally distributed data using a Kruskal−Wallis test. A false discovery rate (*q* value) for multiple testing was calculated using the procedure of Benjamini, Krieger and Yekutieli (Graphpad, USA).

## Supplementary information

Supplementary Material
