# Supplementary material for: Dissecting mechanisms of resistance to targeted drug combination therapy in human colorectal cancer
Source: Oncogene. 2019 Mar 25;38(25):5076–90. doi: 10.1038/s41388-019-0780-z (PMC6755994; doi:10.1038/s41388-019-0780-z)
Supplement: Supplementary file 1 — Supplementary Material [file 41388_2019_780_MOESM1_ESM.docx]

**DISSECTING MECHANISMS OF RESISTANCE TO TARGETED DRUG COMBINATION THERAPY IN HUMAN COLORECTAL CANCER**

Paul A. Clarke, Toby Roe, Kate Swabey, Steve M. Hobbs, Craig McAndrew, Kathy Tomlin, Isaac Westwood, Rosemary Burke, Robert van Montfort and Paul Workman

**SUPPLEMENTARY TABLES AND FIGURES**

**SUPPLEMENTARY TABLES**

**
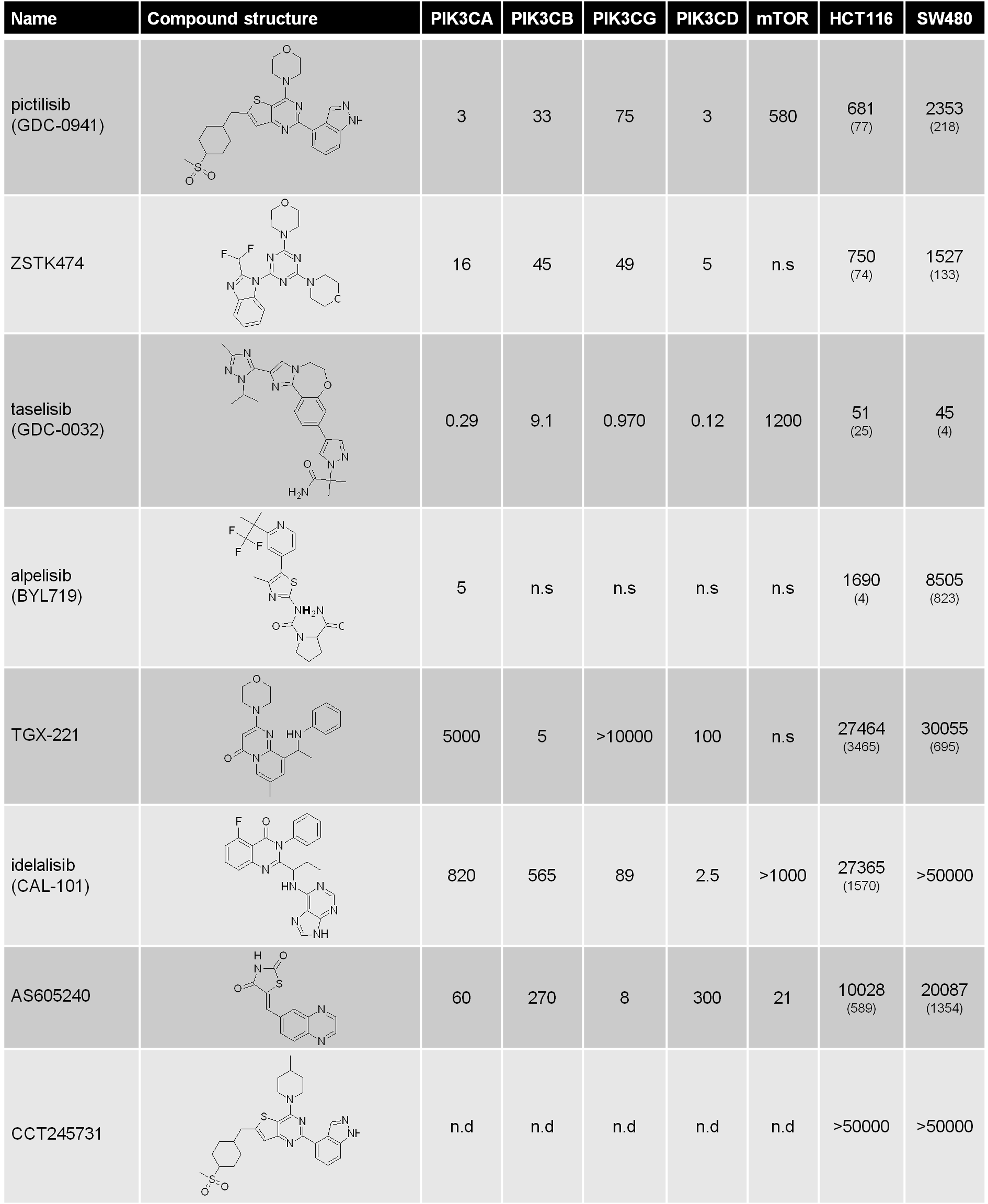
**

**
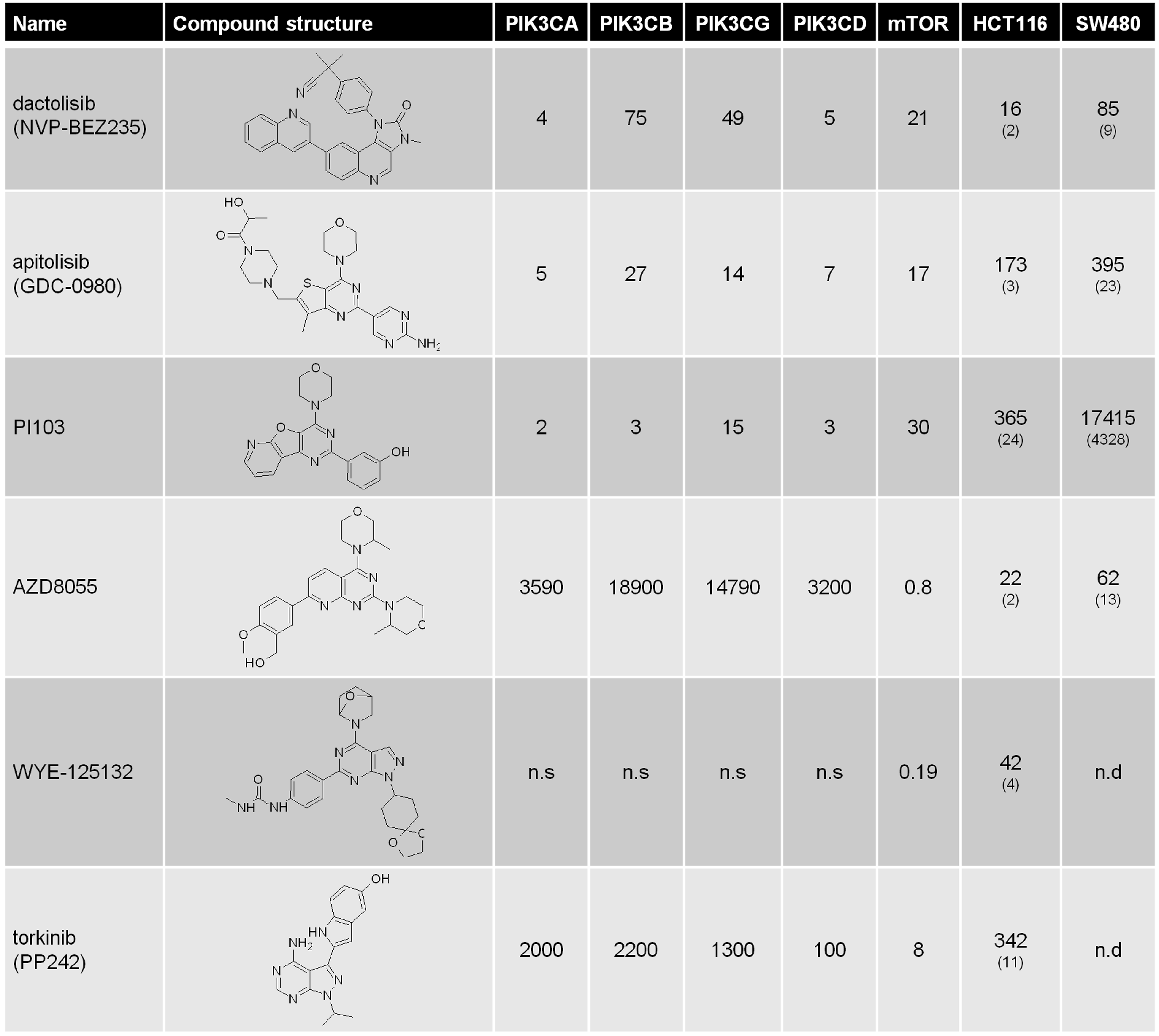
**

**Supplementary Table 1. Structure and activity of PI3K pathway inhibitors.**  GI_50_ determined in-house and K_i_ values (nM) are taken from compound datasheets. GI_50_ values were determined for HCT116 and SW480 human colorectal cancer cell lines following 96 h exposure to each compound (n.d – not determined; n.s – information not supplied on compound datasheet; n = > 3, s.e.m. given in parentheses).

**
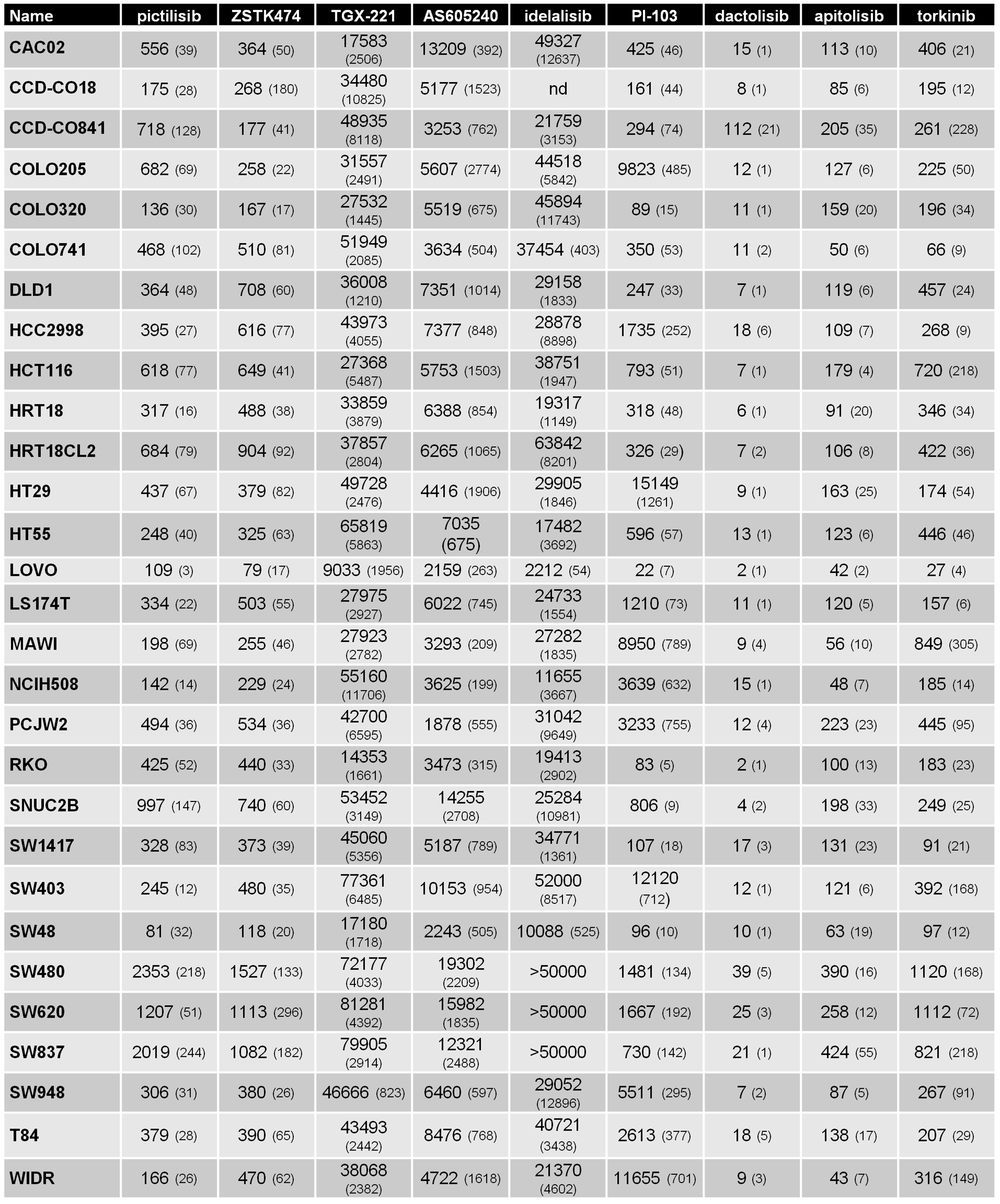
**

**Supplementary Table 2. GI_50_ values for PI3K pathway inhibitors.**  GI_50_ values were determined for human colorectal cancer cell lines following 96 h exposure to each compound (n = > 3, s.e.m. given in parentheses).

**
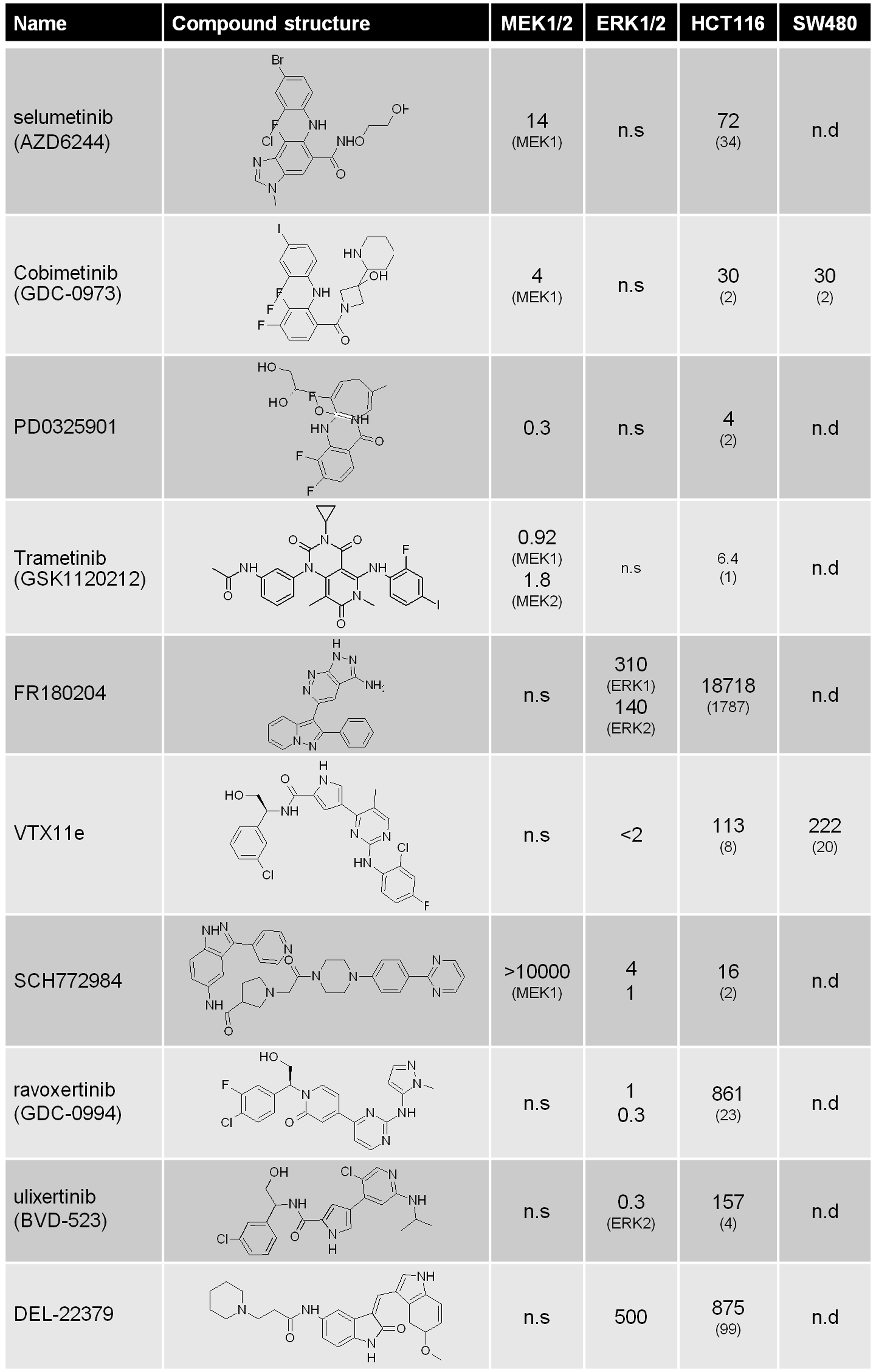
**

**Supplementary Table 3. Structure and activity of MAPK pathway inhibitors.** GI_50_ determined in-house and K_i_ values (nM) are taken from compound datasheets. GI_50_ values were determined for HCT116 and SW480 human colorectal cancer cell lines following 96 h exposure to each compound (n.d – not determined; n.s – information not supplied on compound datasheet; n = > 3, s.e.m. given in parentheses**).**


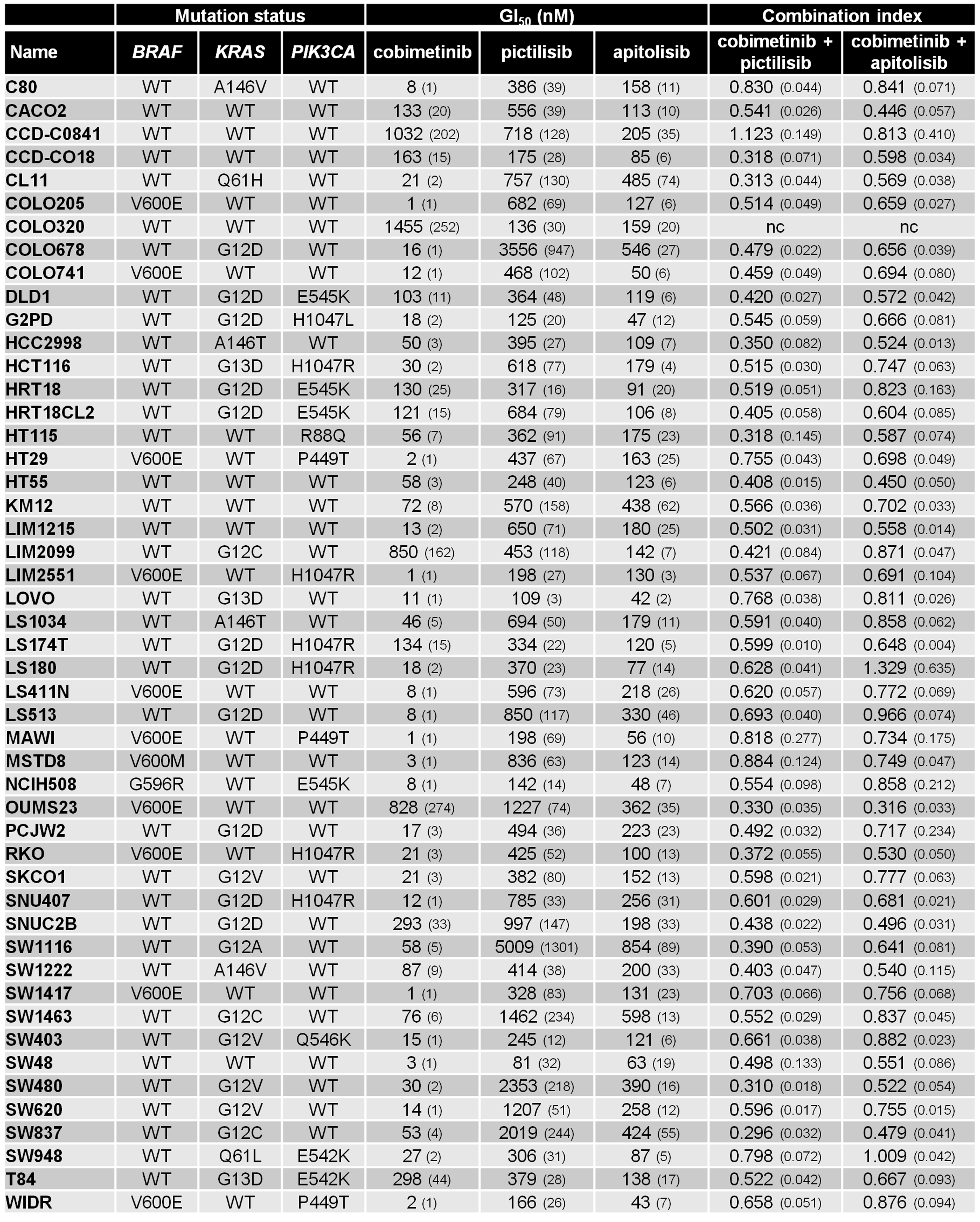


**Supplementary Table 4. GI_50_ and CI values determined for 47 human colorectal cancer lines.**  GI_50_ and CI values were determined for human colorectal cancer cell lines following 96 h exposure to each compound or to combined treatment of pictilisib with cobimetinib or apitolisib (n = > 3, s.e.m. given in parentheses, nc = not calculable).

**
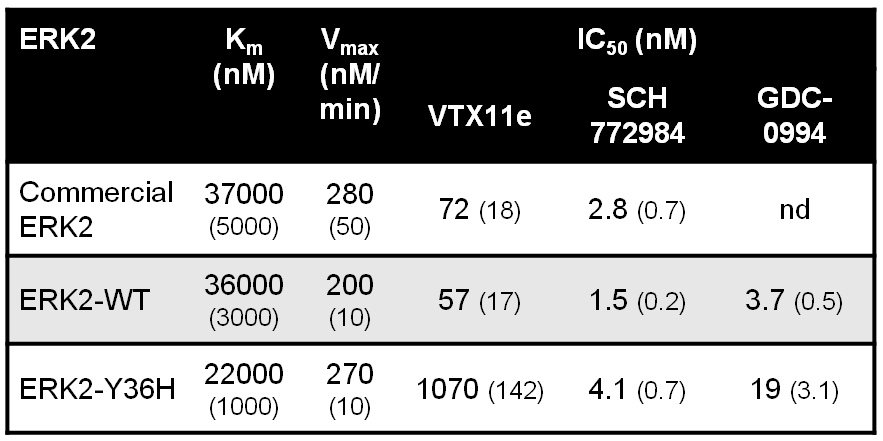
**

**Supplementary Table 5. ERK2^Y36H^ is less sensitive to ERK inhibitors than ERK2^WT^.** Table showing ERK2 enzyme kinetics and inhibitor mean IC_50_ values for ERK2^WT^, ERK^Y36H^ and commercially sourced ERK2 protein as an additional control (n = > 3, s.e.m. given in parentheses; n.d – not determined).

**SUPPLEMENTARY FIGURES**

**
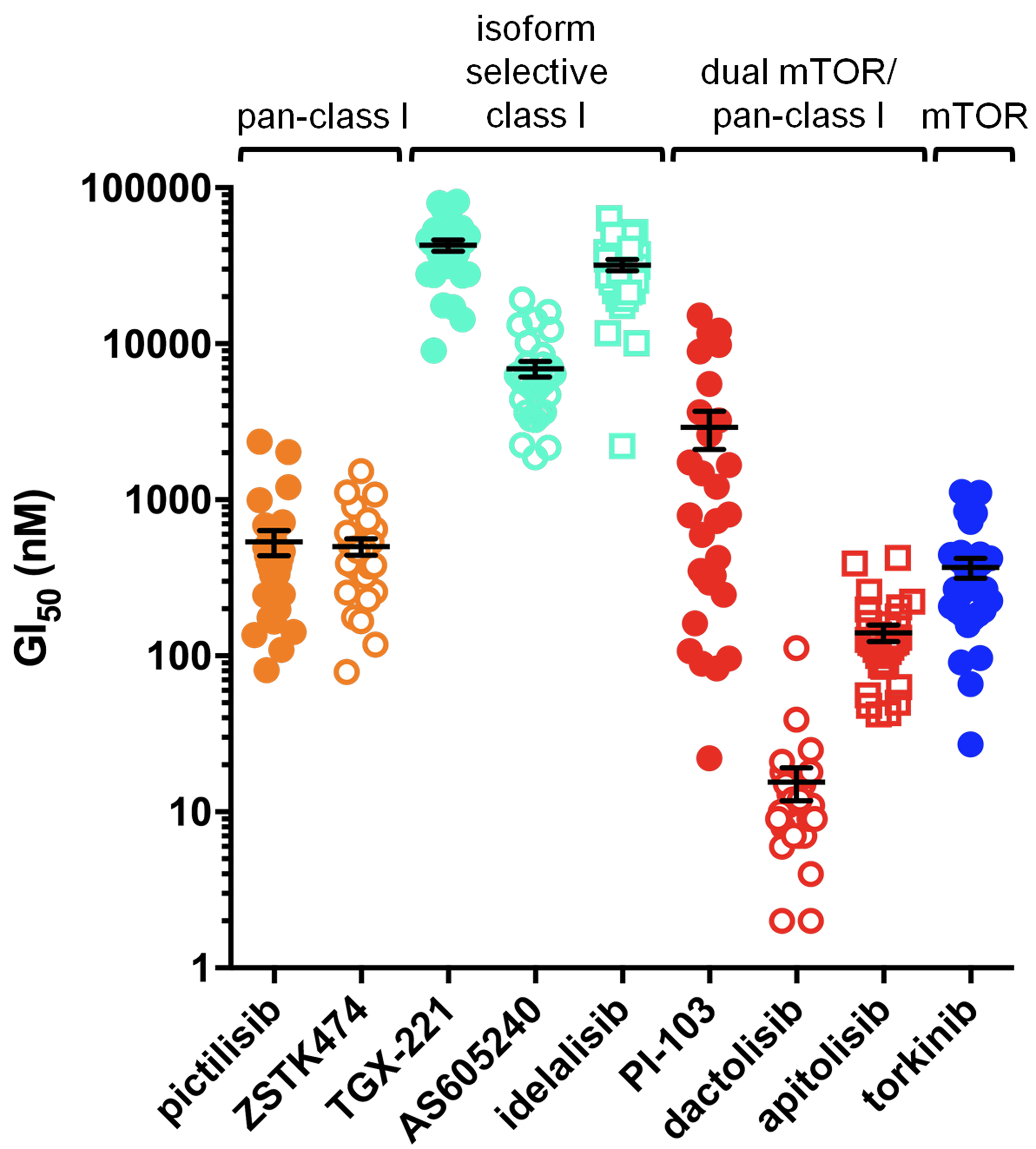
**

**Supplementary Figure 1. Human colorectal cancer cell lines have different sensitivities to PI3K pathway inhibitors with different selectivity profiles.** GI_50_ values for a panel of 29 human colorectal cancer cells. Each data point is a mean value, where n = >3. Mean panel GI_50_ values are superimposed in black (± s.e.m.).

**
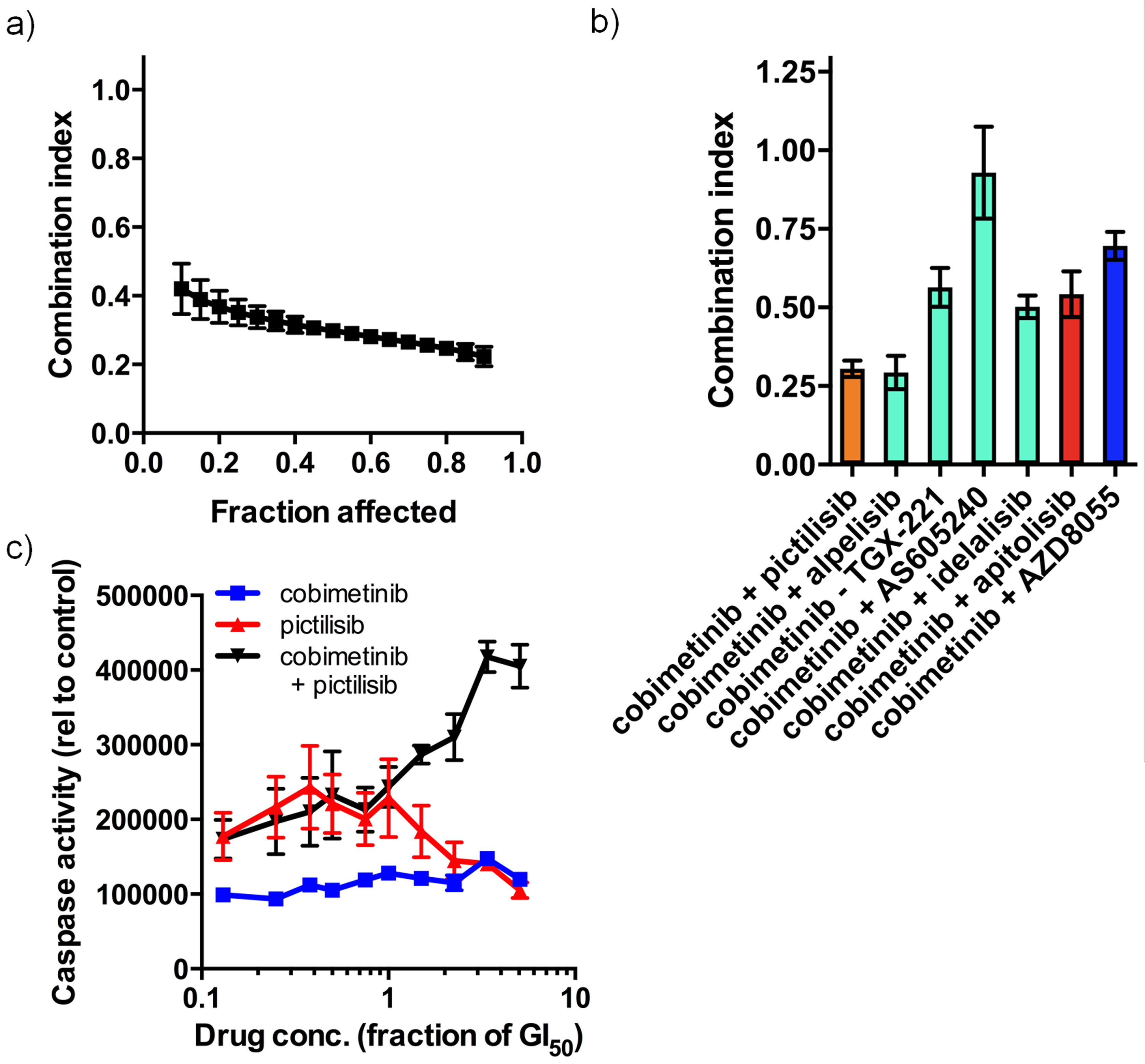
**

**Supplementary Figure 2. MEK and PI3K inhibitors are synergistic for growth inhibition and apoptosis induction in SW480 human colorectal cancer cells.**  (a) Plot of combination index versus fraction affected, after cells were exposed to cobimetinib and pictilisib for 96 h. (b) Combination indices for MEK and PI3K pathway inhibitors with different selectivity profiles (colours indicate PI3K pathway inhibitor selectivity: orange = pan-class I, cyan = class I isoform-selective, red = dual mTOR–pan-class I, blue = mTOR). (c) Caspase 3/7 cleavage, indicative of apoptosis, following 24 h exposure to treatment. For all plots data are mean (s.e.m) values and n = > 3.

**
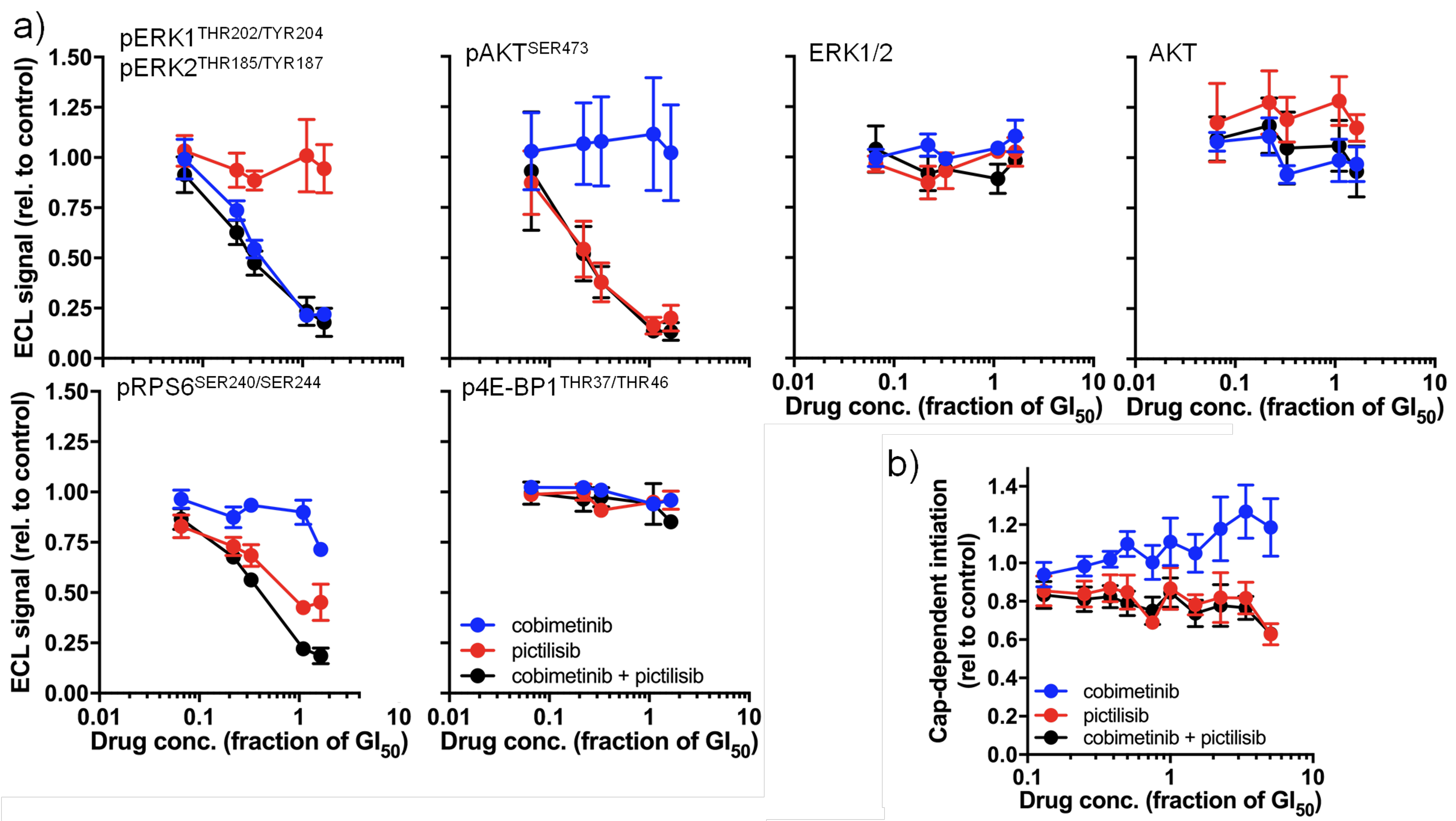
**

**Supplementary Figure 3. Combined MEK–PI3K inhibition has a synergistic effect on RPS6 phosphorylation in SW480 human colorectal cancer cells.** (a) Levels of pERK1^THR202/TYR204^/pERK2^THR185/TYR187^, pAKT^SER473^, ERK1/2, AKT, pRPS6^SER240/SER244^ and p4E-BP1^THR37/THR46^ after cells were exposed to inhibitors for 6 h. (b) Level of cap-dependent protein synthesis initiation after 24 h exposure to cobimetinib and/or pictilisib. Level of cap-dependent protein synthesis initiation (firefly luciferase) after 24 h exposure to cobimetinib and/or pictilisib. Data normalized to cap-independent protein synthesis initiation (EMCV IRES-driven *renilla* luciferase expressed from the same bicistonic mRNA) and expressed relative to vehicle control. All plots show mean values (s.e.m.), n = > 3.

**
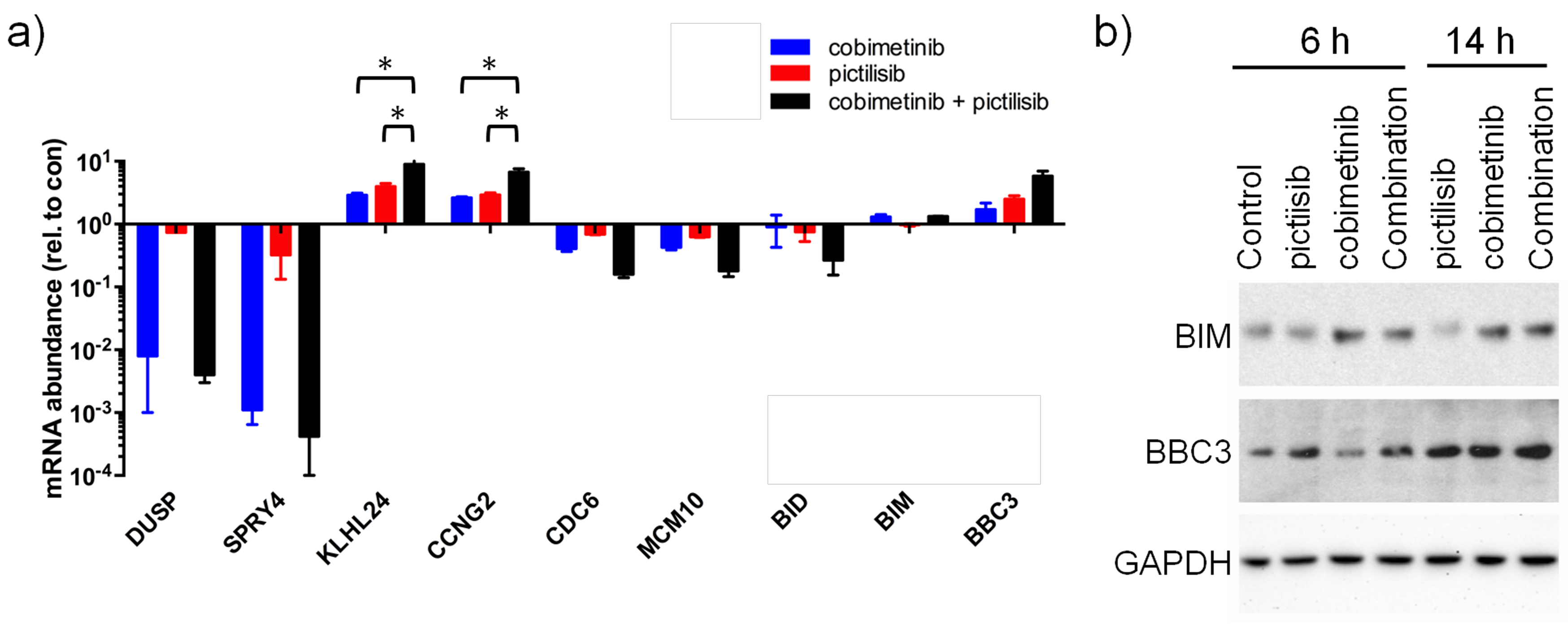
**

**Supplementary Figure 4. Combined MEK–PI3K inhibition increases *KLHL24* and *CCNG2* mRNA and BIM and BBC3 protein levels.**  (a) Transcript abundance in SW480 cells exposed to pictilisib and/or cobimetinib for 6 h, relative to levels in cells treated with a vehicle control (n = > 3; error bars indicate s.e.m; **P* = < 0.001). (b) Immunoblot indicating the abundance of BIM and BBC3 after HCT116 cells were treated with 5 x GI_50_ pictilisib and/or cobimetinib. GAPDH was included as a loading control.

**
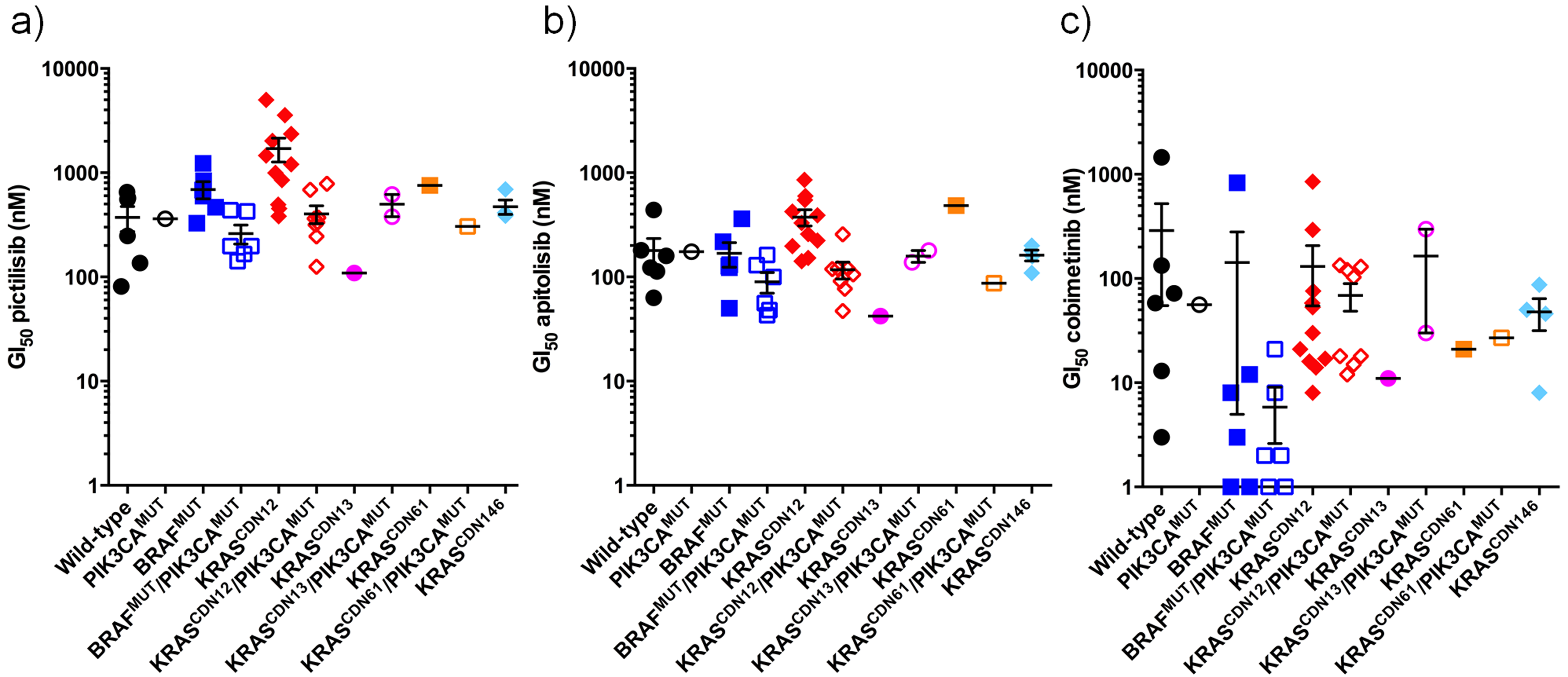
**

**Supplementary Figure 5. *KRAS*^CDN12^ human colorectal cancer cells are less sensitive to PI3K inhibition.** GI_50_ values for **(a)** PI3K inhibitor pictilisib, **(b)** dual mTOR/PI3K inhibitor apitolisib and **(c)** MEK inhibitor cobimetinib across a panel of 47 human colorectal cancer cell lines with different mutation profiles. GI_50_ values were determined after 96 h exposure to the compound (n = > 3). Mean values (s.e.m) are superimposed in black (CDN = codon mutation).

**
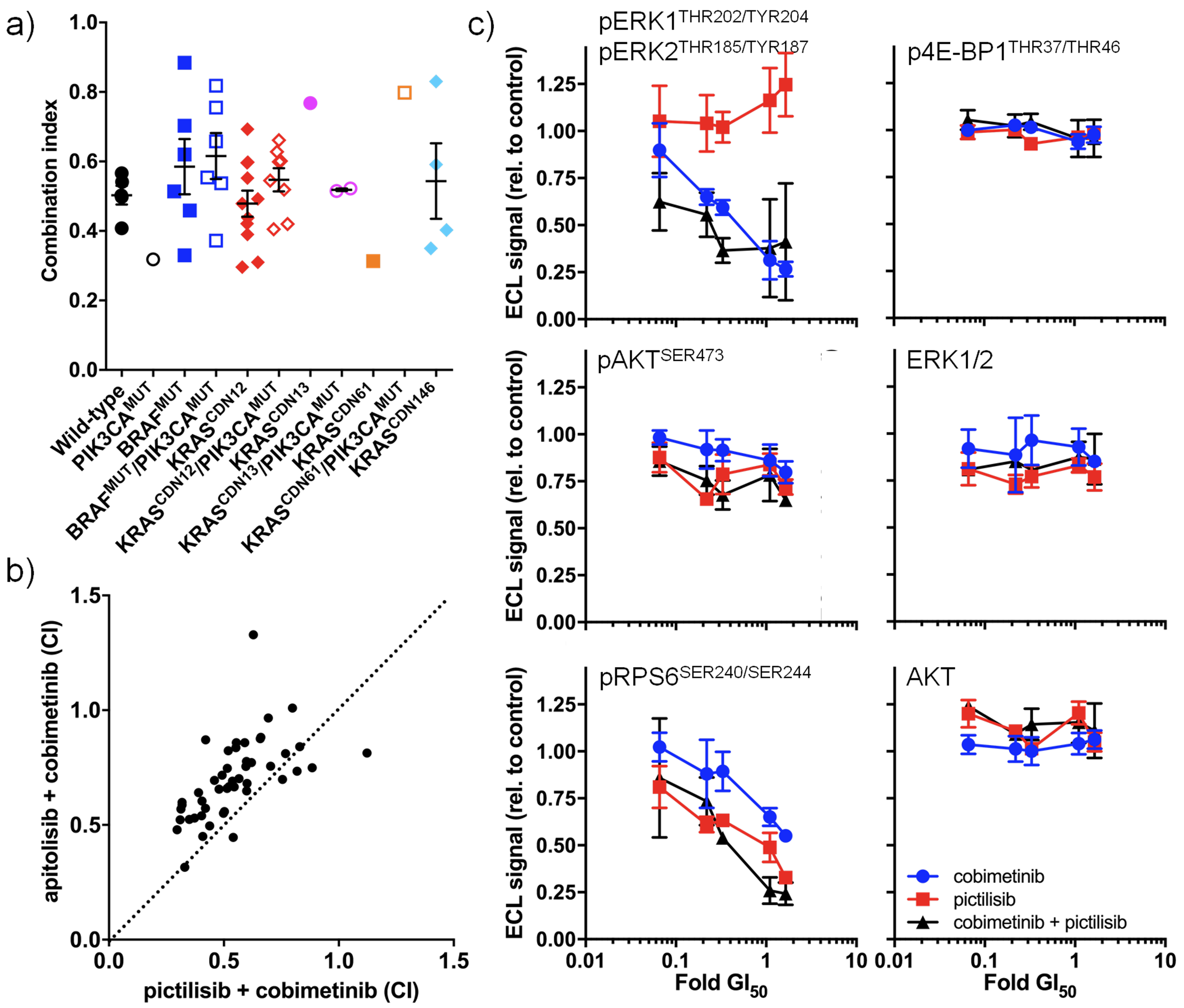
**

**Supplementary Figure 6. Combined MEK–PI3K inhibition is synergistic for cell growth inhibition across a human colorectal cancer panel, but SW620 cells are less sensitive.**  (a) Combination indices for 46 human colorectal cancer cell lines with different *KRAS*/*BRAF*/*PIK3CA* mutation status treated with pictilisib + cobimetinib for 96 h (CDN = codon mutation). (b) Comparison of CI values for cobimetinib + pictilisib versus cobimetinib + apitolisib across a cell line panel. The CI values plotted are at the combination GI_50_. Cells were treated with compounds for 96 h before determining the CI. Dotted line = 1:1 ratio. (c) Mean levels (s.e.m) of pERK1^THR202/TYR204^/pERK2^THR185/TYR187^, pAKT^SER473^, pRPS6^SER240/SER244^, p4E-BP1^THR37/THR46^, ERK1/2 and AKT in SW620 cells exposed to cobimetinib and/or pictilisib for 6 h. Data are plotted relative to levels in cells treated with a vehicle control (n = > 3).

**
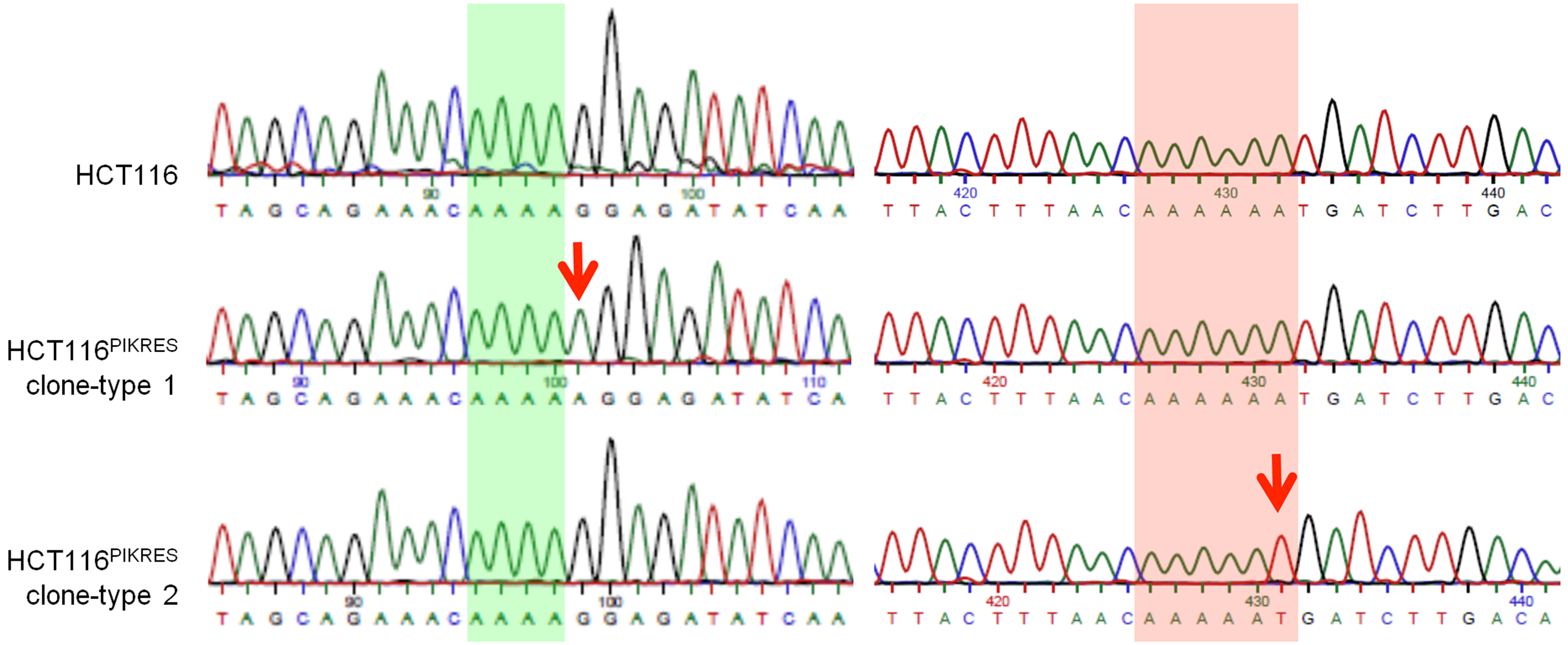
**

**Supplementary Figure 7. HCT116^PI3KRES^ human colorectal cancer cells carry *PTEN* mutations.** Schematic showing the two frameshift mutations detected in *PTEN* cDNA cloned from HCT116^PI3KRES^ cells. Four out of eighteen full-length clones from HCT116^PI3KRES^ cells had the PTEN^K14fs*^ mutation (type 1), fourteen out of eighteen clones carried the PTEN^N323fs*^ mutation (type 2). No wild type *PTEN* cDNA was obtained from HCT116^PI3KRES^ cells; in contrast, all *PTEN* clones from parent HCT116 cells had a wild type sequence.

**
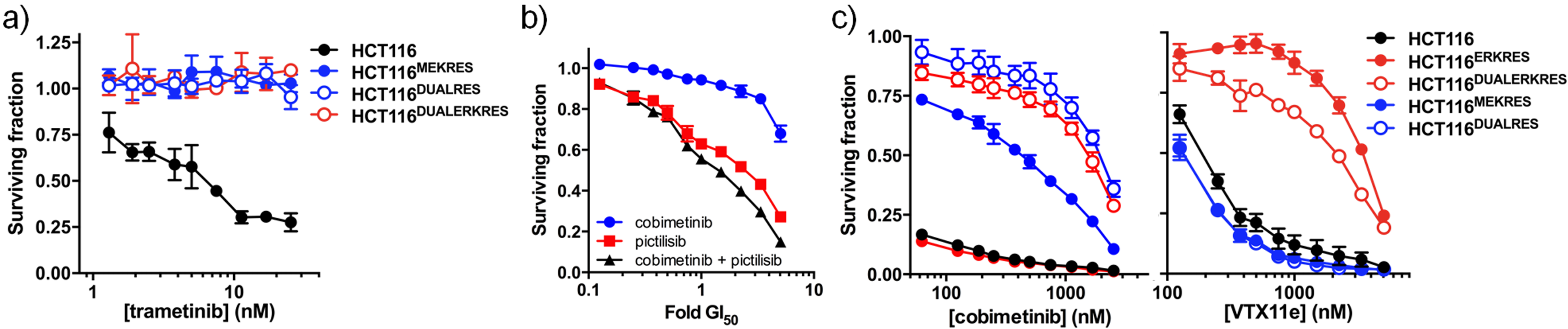
**

**Supplementary Figure 8. The resistance of HCT116 human colorectal cancer cells to PI3K, MEK and ERK inhibitors is dependent on treatment history and cell lineage.** (a) The effect of 96 h exposure to trametinib on the growth of a variety of HCT116 cell lines. (b) The effect on HCT116^DUALERKRES^ cell growth of 96 h exposure to pictilisib and/or cobimetinib. These cells were isolated from HCT116^DUALRES^ cells (resistant to the pictilisib–cobimetinib combination) after continuous culture in medium containing VTX11e and pictilisib. (c) The effect of 96 h exposure to cobimetinib or VTX11e on the growth of a variety of HCT116 cell lines. For all plots data are mean values (s.e.m.), n = > 3.

**
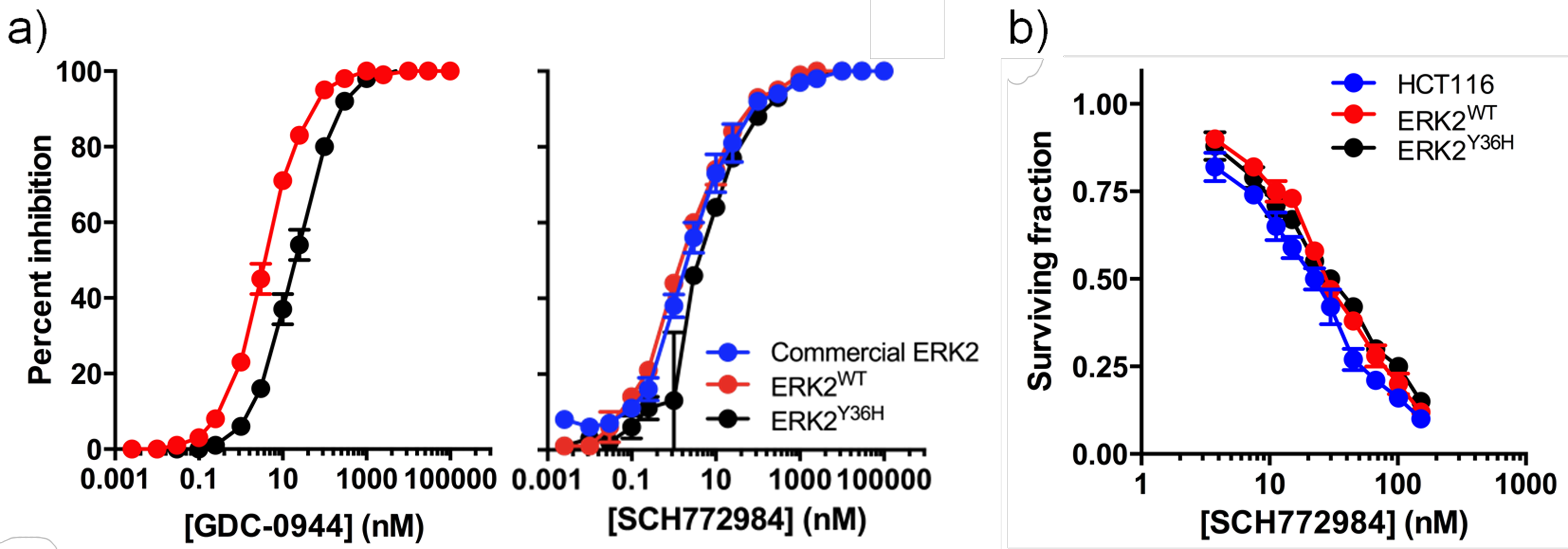
**

**Supplementary Figure 9. ERK2^Y36H^ is less sensitive to ERK inhibitors than ERK2^WT^.**  (a) Inhibition of recombinant ERK2 kinase activity by GDC-0944 or SCH772849. (b) The effect of 96 h treatment with SCH772984 on the growth of HCT116 parent and isogenic ERK2 lines. All plots (a, b) show mean (s.e.m.) values (n = >3).

**
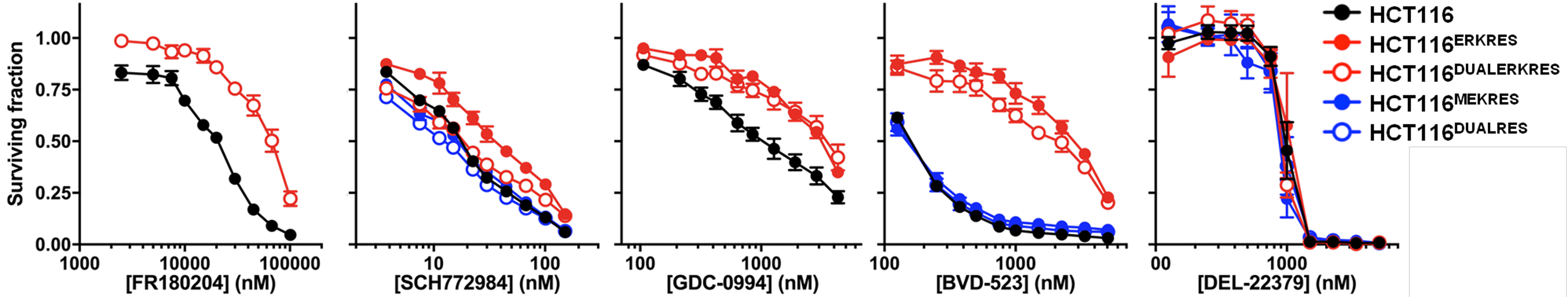
**

**Supplementary Figure 10. Human colorectal cancer HCT116 cells with an acquired ERK2^Y36H^ mutation are less sensitive to some ERK inhibitors.** Cells were treated for 96 h with test compounds (Supplementary Table 3). All plots are of mean data (s.e.m), where n = > 3**.**

**
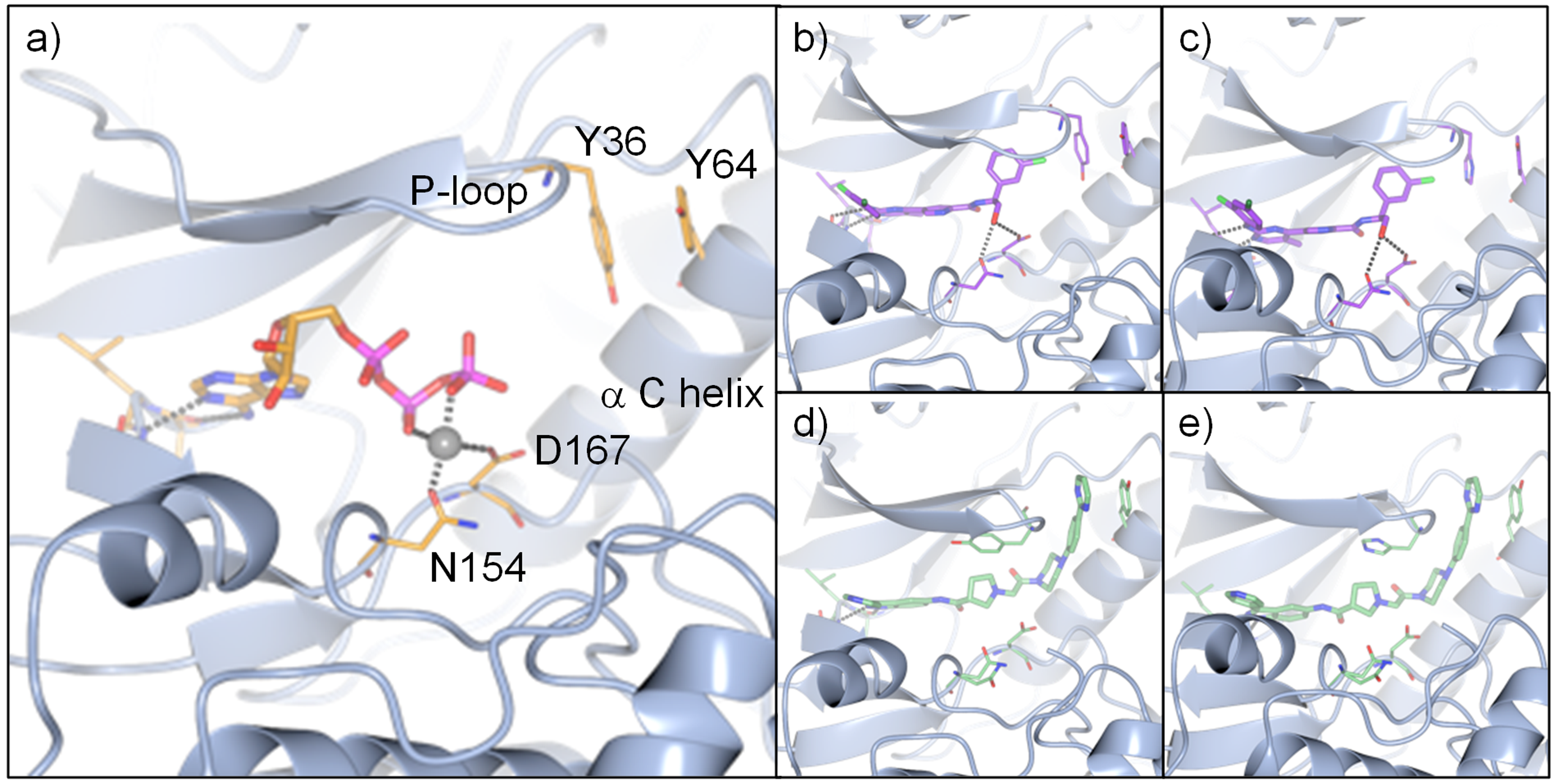
**

**Supplementary Figure 11. The ERK2^Y36H^ mutation hinders VTX11e binding.** The impact of the ERK2^Y36H^ mutation was modelled onto existing ERK2 crystallography structures. (a) Rat ERK2 in complex with ATP (PDB code 4GT3) showing a magnesium ion (grey) mediating β- and γ-phosphate binding to the DFG aspartic acid D167 and to N154 (human numbering scheme). Y36 and Y64 form a parallel-displaced π-π interaction between the tip of the P-loop and the αC helix. (b) Human ERK2 in complex with VTX11e (PDB code 4QTE).^1^ VTX11e binds in the same orientation as ATP. The hydroxymethyl group mimics the magnesium ion, forming H bonds to D167 and N154. The chlorophenyl group packs underneath the P-loop and directs the Cl atom towards the π-cloud of Y36. (c) Predicted structure of human ERK2^Y36H^ in complex with VTX11e. A larger closure of the N-lobe is required to position the smaller, more electron-rich H36 side chain. The imidazole ring of H36 is predicted to repel the partial negative charge on the aryl chloride of VTX11e. (d) Human ERK2 in complex with SCH772984 (PDB code 4QTA).^1^ Y36 folds underneath the P-loop, making a hydrophobic interaction with the pyrrolidine moiety of SCH772984. The pyrimidine group of the ligand π-stacks with Y64. (e) Predicted structure of ERK2^Y36H^ in complex with SCH772984. Binding is not predicted to alter dramatically: H36 would form hydrophobic interactions with the pyrrolidine of the ligand, maintaining the binding mode and protein fold observed with the WT structure.


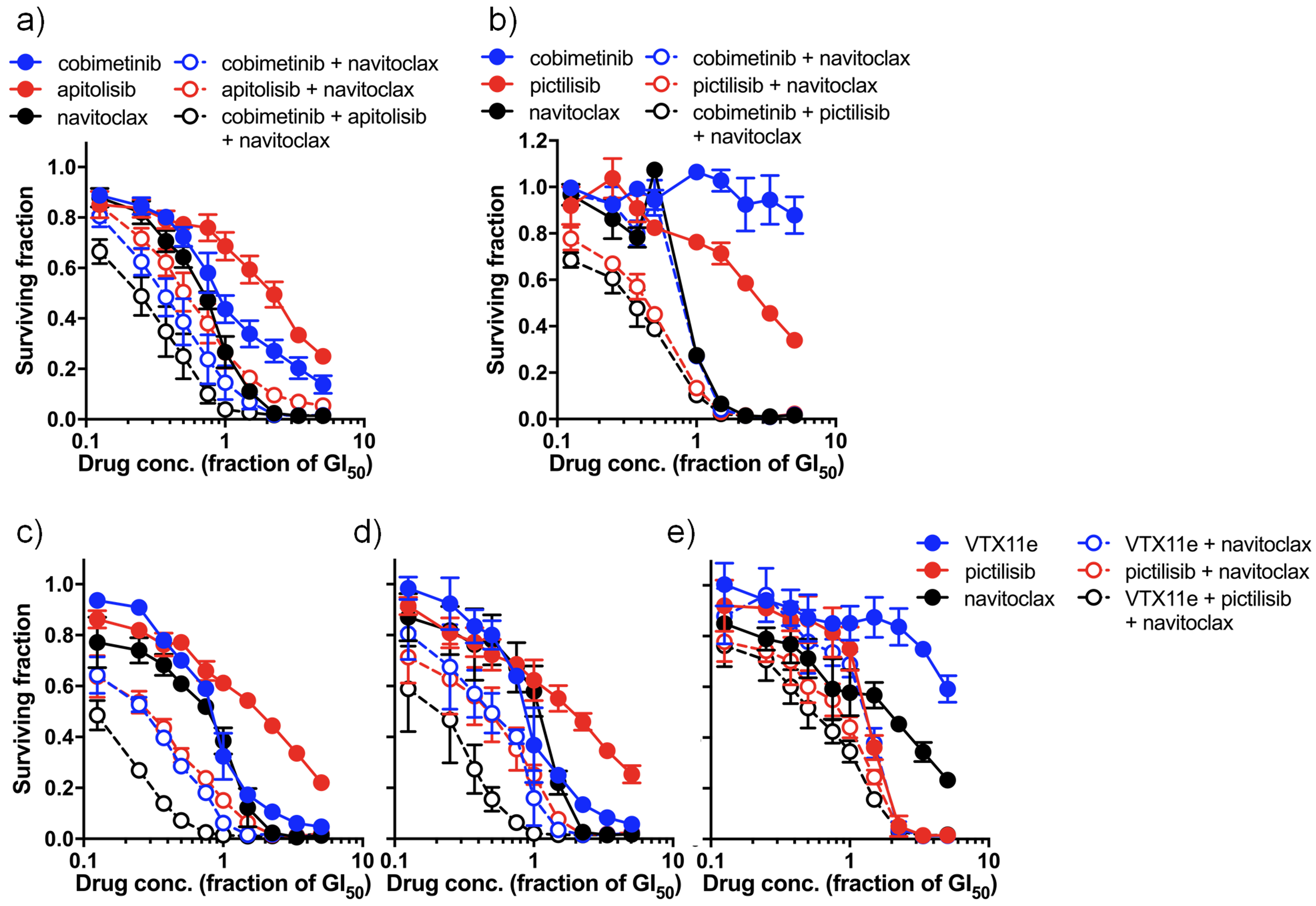


**Supplementary Figure 12. Cells with previously acquired resistance to two-way combinations are less responsive to three-way combinations.** Three-way combination treatment of parent or combination resistance HCT116 cells following 96 hours exposure to cobimetinib or VTX11e, pictilisib or apitolisib and navitoclax at 1:1:1 ratios of their respective GI_50_s**.** (a) HCT116^DUALRES^ cells treated with cobimetinib, apitolisib and navitoclax, (b) HCT116^DUALRES^ cells treated with cobimetinib, pictilisib and navitoclax and (c) parent HCT116, (d) HCT116^DUALRES^ and (e) HCT116^DUALERKRES^ cells treated with VTX11e, pictilisib and navitoclax. For all plots data are mean values (s.e.m.), n = > 3.

**REFERENCES**

1 Chaikuad A, Tacconi EMC, Zimmer J, Liang Y, Gray NS, Tarsounas M *et al.* A unique inhibitor binding site in ERK1/2 is associated with slow binding kinetics. *Nat Chem Biol* 2014; **10**: 853–860.
